# Mechanism Investigation of *Tagetes patula* L. against Chronic Nonbacterial Prostatitis by Metabolomics and Network Pharmacology

**DOI:** 10.3390/molecules24122266

**Published:** 2019-06-18

**Authors:** Xueying Liu, Xiaoku Ran, Muhammad Riaz, Haixue Kuang, Deqiang Dou, Decheng Cai

**Affiliations:** 1 College of Pharmacy, Liaoning University of Traditional Chinese Medicine, Dalian 116600, China; liuxueying0806@126.com (X.L.); xkran@163.com (X.R.); pharmariaz@outlook.com (M.R.); 2 Department of Pharmacy, Shaheed Benazir Bhutto Sheringal Dir Upper, Khyber Pakhtoon Khwa 18000, Pakistan; 3 College of Pharmacy, Heilongjiang University of Chinese Medicine, Harbin 150040, China; 4 Dalian Wuzhou Holy Herb Scientific and Techonological Co. Ltd, Dalian 116600, China; caidecheng1@126.com

**Keywords:** *Tagetes patula* L., chronic nonbacterial prostatitis, metabolomics, energy metabolism, network pharmacology

## Abstract

The major objective of this study was to investigate the anti-chronic nonbacterial prostatitis (CNP) mechanism of *T. patula* by metabolomics and network pharmacology. The study demonstrated that the flavonoids and polysaccharides of *T. patula* could alleviate prostatitis by improving the level of DHT, reducing the secretion of PSA and TNF-α. Besides, both could enhance Na^+^/K^+^-ATPase activity, decrease the O_2_ consumption, CO_2_ production, heat production, energy expenditure of rats and promote respiratory exchange ratio of rats. Up to 28 potential biomarkers and 8 key metabolic pathways related to the treatment of CNP were elucidated by the metabolomics analysis, including phenylalanine metabolism, taurine and hypotaurine metabolism, tryptophan metabolism etc. Network pharmacology prediction also reflected the potential mechanism was associated with tryptophan metabolism and energy pathway. Generally, the potential anti-CNP mechanism of flavonoids and polysaccharides of *T. patula* might be through reducing the expression of inflammation factors, adjusting the level of hormone and regulating the amino acid metabolism, energy metabolism and glucose and lipid metabolism.

## 1. Introduction

Prostatitis is a common urinary system disease that endangers the health of adult males. Chronic nonbacterial prostatitis (CNP), as the most common type of prostatitis, is difficult to cure and easily recurs though there are several methods for treatment. *T. patula* is a perennial herb belonging to the family of Asteraceae, commonly known as French marigold, and it is also a traditional drug in Argentina, where it is used for the diuretic agent associated with prostatitis treatments [1,2]. We had illustrated that flavonoids were the main components of *T. patula* (Supplementary Materials Figure S1), and previous study had investigated that flavonoids and polysaccharides of *T. patula* were the effective constituents against CNP and their efficacy might be associated with hormone and the inflammatory mediators [3]. The emergence of metabolomics provided a new strategy to investigate the action mechanisms of *T. patula* anti-CNP. As a newly developed strategy, network pharmacology focused on searching for the relationships of active ingredients and their potential targets, which might be associated with pharmacological mechanisms in metabolomics. Therefore, this research aimed at deeply investigating the pharmacological mechanism of active constituents of *T. patula* against CNP through metabolomics and network pharmacology. 

## 2. Results

### 2.1. The Levels of Physiological Indexes

**Figure 1:**
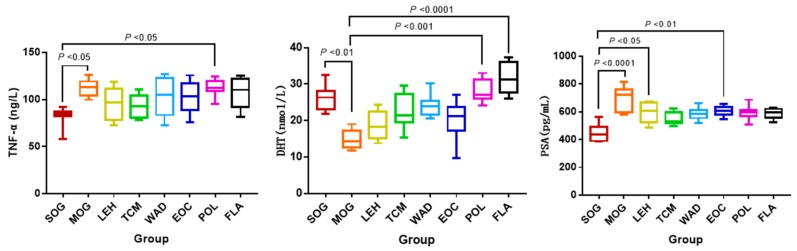
The concentrations of tumor necrosis factor-α (TNF-α), dihydrotestosterone (DHT) and prostate-specific antigen (PSA). Notes: Sham-operated group (SOG), Chronic nonbacterial prostatitis model group (MOG), Water decoction treatment group (WAD), Essential oil treatment group (EOC), Polysaccharide treatment group (POL), Flavonoids treatment group (FLA), *Pule’an* tablet treatment group (TCM), Levofloxacin treatment group (LEH).

Compared with the Sham-operated group (SOG), the levels of prostate-specific antigen (PSA) and tumor necrosis factor-α (TNF-α) in the chronic nonbacterial prostatitis model group (MOG) were a significant increase (*p* < 0.05, *p* < 0.001), and the levels of dihydrotestosterone (DHT) in MOG decreased obviously (*p* < 0.01). Serum DHT level was significantly higher in the polysaccharide treatment group (POL) and flavonoids treatment group (FLA) than in MOG (*p* < 0.001, *p* < 0.0001). Comparing with MOG, the serum PSA level in *Pule’an* tablet treatment group (TCM); Water decoction treatment group (WAD), POL and FLA all showed decreasing trends, and the content of TNF-α in levofloxacin treatment group (LEH), TCM, WAD, EOC and FLA also showed decreasing trends (Figure 1). The histological results of the prostate were shown in Supplementary Materials Figure S4: Histological analysis of prostate.

### 2.2. The Activity of Na^+^/K^+^-ATPase

**Figure 2:**
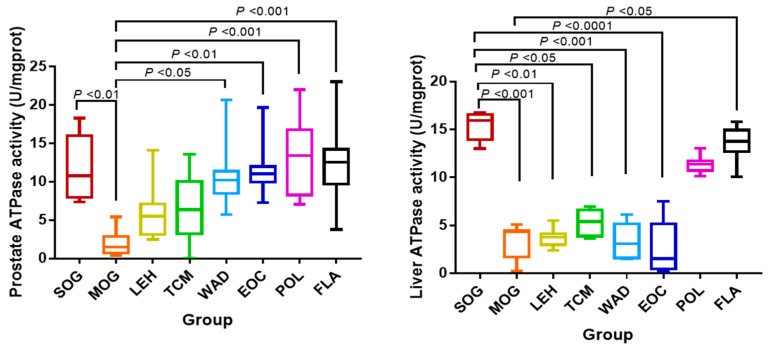
The activities of Na^+^/K^+^-ATPase in prostate and liver tissues. Notes: Sham-operated group (SOG), Chronic nonbacterial prostatitis model group (MOG), Water decoction treatment group (WAD), Essential oil treatment group (EOC), Polysaccharide treatment group (POL), Flavonoids treatment group (FLA), *Pule’an* tablet treatment group (TCM), Levofloxacin treatment group (LEH).

Comparing with SOG, Na^+^/K^+^-ATPase activity in MOG prostate was significantly reduced (*p* < 0.01). And comparing with MOG, water decoction and all the components of *T. patula* could enhance the Na^+^/K^+^-ATPase activity of prostate, in which POL and FLA were significantly increased extremely (*p* < 0.001) and WAD and EOC was increased significantly (*p* < 0.05, *p* < 0.01). Comparing with SOG, Na^+^/K^+^-ATPase activity of livers in MOG, LEH, TCM, WAD and EOC were substantially decreased (*p* < 0.001, *p* < 0.01, *p* < 0.05, *p* < 0.001, *p* < 0.0001). Na^+^/K^+^-ATPase activity of liver in FLA was significantly stronger than in MOG (*p* < 0.05) (Figure 2).

### 2.3. Results of Energy Metabolic Parameters 

**Figure 3:**
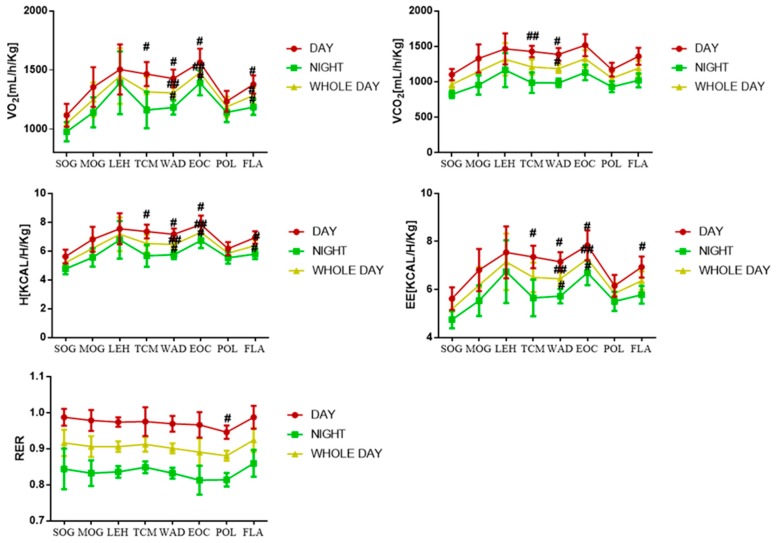
Changes of VO_2_, VCO_2_, H, EE and RER in rats during the day, night and the whole day. Notes: ^#^ *p* < 0.05, ^##^ *p* < 0.01 compared with SOG. Sham-operated group (SOG), Chronic nonbacterial prostatitis model group (MOG), Water decoction treatment group (WAD), Essential oil treatment group (EOC), Polysaccharide treatment group (POL), Flavonoids treatment group (FLA), *Pule’an* tablet treatment group (TCM), Levofloxacin treatment group (LEH), O_2_ consumption (VO_2_), CO_2_ production (VCO_2_), Heat production (H), Energy expenditure (EE), Respiratory exchange ratio (RER).

Based on actual animal weight calculations, comparing with SOG, the O_2_ consumption (VO_2)_, CO_2_ production (VCO_2_), heat production (H) and energy expenditure (EE) in MOG and other treatment groups all show increasing trends no matter when the time was. However, energy metabolism parameters of WAD, POL and FLA all showed a decline at a certain degree. Respiratory exchange ratio (RER) of MOG and other treatment groups showed downtrends in comparison with SOG. From Figure 3, a difference between the model rats and sham rats in terms of energy metabolism can be seen.

### 2.4. LC-MS Analysis of Metabolic Profiling

**Figure 4:**
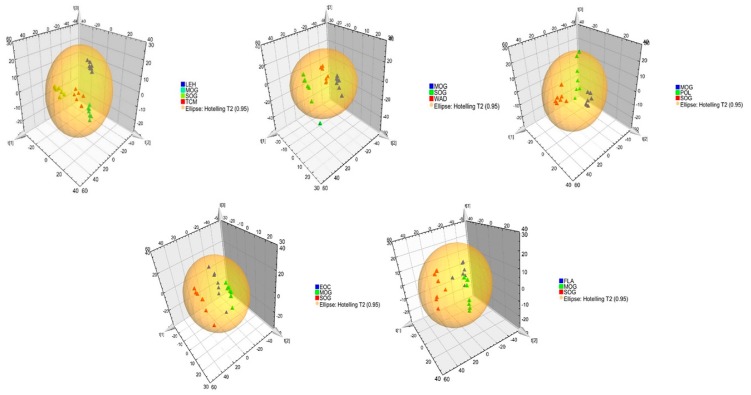
3D PLS-DA score plot in positive ion mode. Notes: Sham-operated group (SOG), Chronic nonbacterial prostatitis model group (MOG), Water decoction treatment group (WAD), Essential oil treatment group (EOC), Polysaccharide treatment group (POL), Flavonoids treatment group (FLA), *Pule’an* tablet treatment group (TCM), Levofloxacin treatment group (LEH).

Using the super-high performance liquid chromatography-mass spectrum in series (UPLC-MS) conditions described in the methods, the representative total ion current (TIC) chromatograms of urine samples of different groups which harvested by UPLC/ electrospray ionization quadrupole-time of flight (ESI-Q-TOF)/MS analysis were presented in Supplementary Materials Figure S2: TIC chromatograms on positive ion mode and Figure S3: TIC chromatograms on negative ion mode. The contours of these spectra differed between 3–5 min and 8–10 min in positive and negative TIC. The PCA and PLS-DA results displayed as score plots showed the scatter of the samples, which indicated similar metabolomics compositions when clustered together and compositional difference metabolomes when dispersed. Overall view of all PCA score plots, SOG and MOG were significantly divided into two classes, indicating that the model of CNP was successfully reproduced. As a supervised pattern recognition method, PLS-DA could better reflect the difference of distinct groups. The corresponding PLS-DA score plots also indicated that MOG is clearly separated from SOG, which implied that the metabolic characteristics of the various small molecules are obviously different. The 3D of PLS-DA score plot shows that SOG, MOG, POL and FLA were separated clearly, and the POL and FLA group was closer to SOG than MOG, which suggested that polysaccharides and flavonoids could reverse the pathological process of CNP (Figure 4). 

### 2.5. Identification of Metabolite Candidates

All collected samples were analyzed. Targeted and non-targeted metabolite candidates were identified as above described methods. Targeted metabolomics identified a total of 13 metabolites and non-targeted metabolomics provided 94 metabolites. A total of 28 metabolite candidates were designated by comprehensively comparative analysis of VIP and *P* values with significant differences (Table 1).

### 2.6. Pathway Analysis

**Figure 5:**
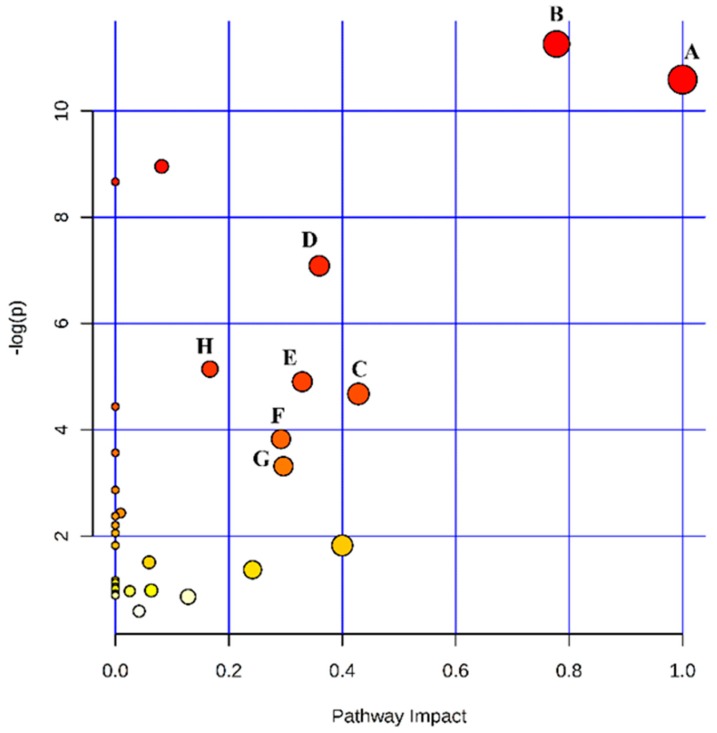
Summary of pathway analysis. Notes: A. Phenylalanine, tyrosine and tryptophan biosynthesis; B. Phenylalanine metabolism; C. Taurine and hypotaurine metabolism; D. Tryptophan metabolism; E. Tyrosine metabolism; F. Glyoxylate and dicarboxylate metabolism; G. Glycine, serine and threonine metabolism; H. Citrate cycle (TCA cycle).

To gain insight into the metabolic mechanism of CNP, metabolic pathways of the significantly altered metabolites were analyzed by using the pathway analysis module within the Masslynx V4.1 Workstations. Related pathways of biomarkers were identified by searching Kyoto Encyclopedia of Genes and Genomes (KEGG) and Human Metabolome Database (HMDB) PATHWAY Database. We identified a total of 31 metabolic pathways which were related to the metabolite candidates (Table 2). However, only 8 distinct metabolic pathways were significantly altered in the urine samples as compared with the model group (*p* < 0.05, impact > 0.1). The predominant hits were pathways involved in phenylalanine, tyrosine and tryptophan biosynthesis, phenylalanine metabolism, taurine and hypotaurine metabolism, tryptophan metabolism, tyrosine metabolism, glyoxylate and dicarboxylate metabolism, glycine, serine and threonine metabolism and citrate cycle (TCA cycle) (Figure 5). Results indicated that these pathways showed the marked perturbations over the formation of CNP and could contribute to the development of CNP.

### 2.7. Network Pharmacology

**Figure 6:**
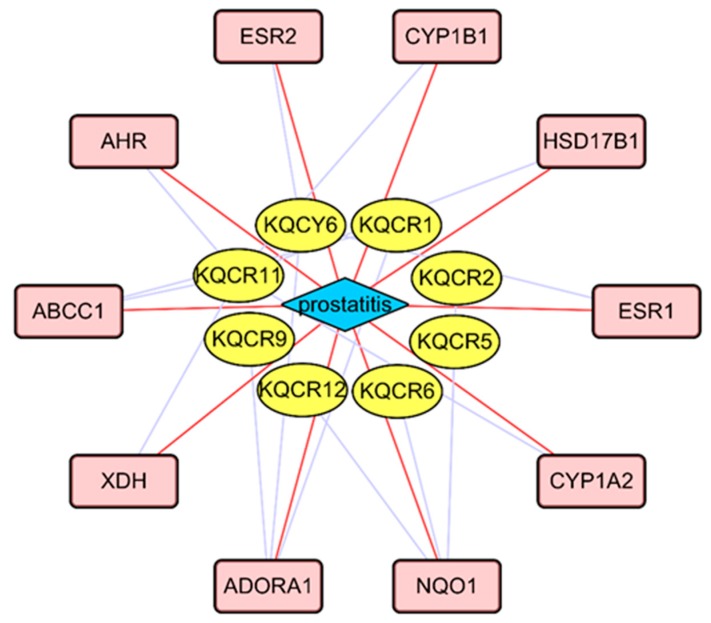
*T. patula* active compounds-target-prostatitis disease interaction.

**Figure 7:**
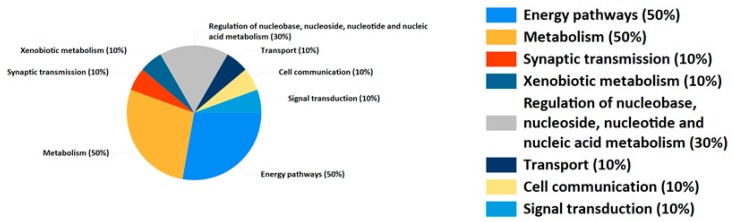
Biological process for merge gene.

**Figure 8:**
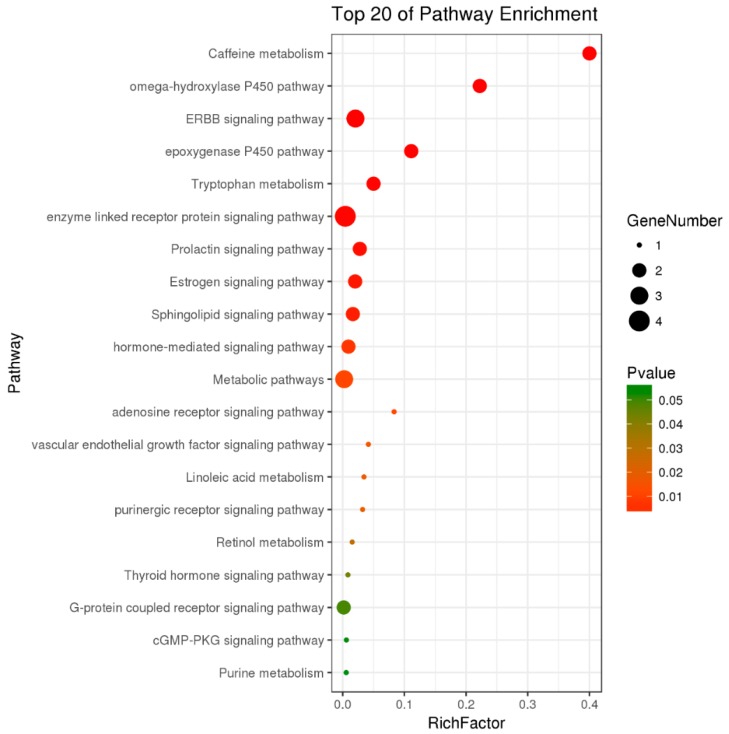
Pathway enrichment analysis of network pharmacology.

Figure 6 illustrates the interaction between the active compounds in *T. patula* and a potential target for prostatitis disease. In total, this network comprised 19 nodes (8 active compounds, 1 disease and 10 potential drug targets) and 25 edges. From Figure 6, we found that various compounds could hit multiple potential targets, while some could only hit less potential biomarkers. Compounds that can hit multiple potential targets were thought to be major active compounds in anti-prostatitis. Functional analysis by gene enrichment function of FunRich software indicated that up to 50% merge genes were associated with energy pathway and metabolism (Figure 7). And the results of enriched pathways showed that caffeine metabolism, omega-hydroxylase P450 pathway, ERBB signaling pathway, epoxygenase P450 pathway and tryptophan metabolism had more richfactor (Figure 8). The number of genes in RichFactor, P value, and enrichment pathways is a measure of KEGG enrichment. The larger the RichFactor, the greater the degree of enrichment is. The value of *p* value is [0,1], and the closer to 0, the more significant the enrichment is. Potential targets of active compounds and relevant target genes of prostatitis were shown in Supplementary Materials Table S1: Potential targets of active compounds. and Table S2: Relevant target genes of prostatitis. Information of merging gene was displayed in Supplementary Materials Table S3: The information of merging gene. Details of enriched pathways are summarized in the Supplementary Materials Table S4: Details of enriched pathways.

## 3. Discussion

As is well known, the main manifestations of prostatitis exhibited a pain in the pelvic region and dysfunction of urination. The main manifestations of pain exhibited anxiety, tachypnea, hypertension, hyperhidrosis and etc. It is interesting to note that the increasing of VO_2_ and VCO_2_ in this study might be caused by tachypnea, so as to promote the degree of H and EE and decrease RER. However, the flavonoids constituent and polysaccharides constituent of *T. patula* could reverse those. In addition, the increasing of Na^+^/K^+^-ATPase’s activities in prostate and liver also reflected that the flavonoids and polysaccharides of *T. patula* could regulate energy metabolism.

Urine samples were analyzed by UPLC/ESI-TOF-MS and multivariate statistical analysis. The results showed that the area of dynamic metabolic profiles after polysaccharide and flavonoids treatment was close to the sham-operated group, demonstrating that polysaccharide and flavonoids of *T. patula* had therapeutic efficacy. Metabonomics analysis discovered that phenylalanine, tyrosine and tryptophan biosynthesis and phenylalanine metabolism in the rat prostatitis model showed abnormal metabolism. Compared with SOG, the levels of l-tyrosine, phenylacetaldehyde, phenylpyruvic acid, l-phenylalanine, pantetheine, l-cysteine, pantothenic acid, serotonin, l-kynurenine, 4-(2-Aminophenyl)-2,4-dioxobutanoic acid, 3-indoleacetic acid, d-xylulose, l-histidine, epinephrine, maleylacetoacetic acid, taurine, glycine, uric acid and adenine in the urine of model rats were reduced to varying degrees, and the contents of uracil, tryptophan, indoleacrylic acid, d-glucuronic acid, 2-oxoglutarate, l-malic acid, citrate, dopamine and l-threonine in the urine of model rats were increased to different degrees. After the treatment of polysaccharide and flavonoids of *T. patula*, the concentration of l-tyrosine, l-phenylalanine, pantetheine, l-cysteine, pantothenic acid, 4-(2-Aminophenyl)-2,4-dioxobutanoic acid, 3-indoleacetic acid, l-histidine, maleylacetoacetic acid, taurine, glycine, uric acid, adenine, tryptophan, indoleacrylic acid, d-glucuronic acid, 2-oxoglutarate, l-malic acid, citrate and l-threonine in urine were adjusted backwards to normal in certain degrees.

These above differential metabolite identification results referred the pathway related to phenylalanine, tyrosine and tryptophan biosynthesis, phenylalanine metabolism, pantothenate and CoA biosynthesis, tryptophan metabolism, pentose and glucuronate interconversions, aminoacyl-tRNA biosynthesis and taurine and hypotaurine metabolism. These disturbed metabolic pathways can be partially reversed by the polysaccharides and the total flavonoids of *T. patula*, in other words, they may help in repairing these metabolites.

**Figure 9:**
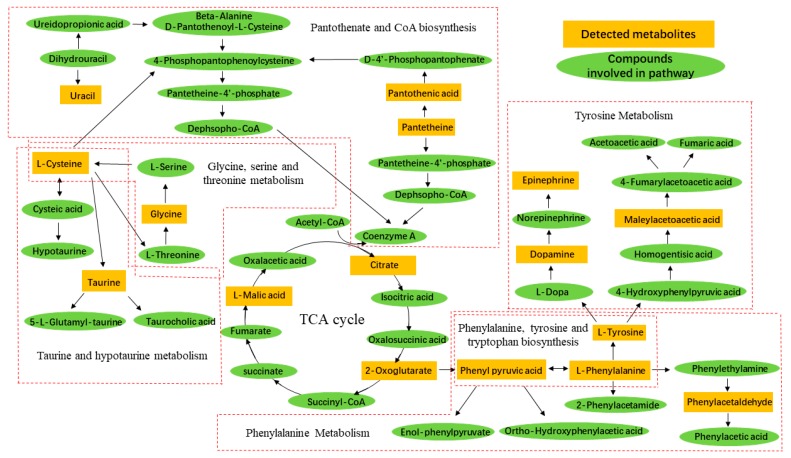
Related metabolic pathway.

Network pharmacology results also reflect the potential mechanism of active compounds in *T. patula* anti-prostatitis. The analysis results showed that the compounds in *T. patula* acts on multiple targets to anti-prostatitis. By integrating the metabolomics pathway and the target pathways predicted by network pharmacology, we found that they are similar, which could explain the accuracy of the predicted target. Research indicated that prostatitis is associated closely with hormone and inflammatory level. Uric acid has the antioxidant capacity and can prevent the emergence of an inflammatory environment [4,5,6,7]. Generally, the androgen of prostatitis patients is lower than healthy people. Amino acids are not merely the constituent units of proteins, but also can participate in hormone biosynthesis [8,9]. l-phenylalanine is the precursor of the tyrosine and catecholamines, while tyrosine is closely linked to the formation of certain hormones and neurotransmitters such as dopamine and epinephrine [10]. Phenylpyruvic acid and phenylacetaldehyde both are the intermediate or catabolic byproduct of phenylalanine metabolism [7]. Dopamine is a precursor to adrenaline and norepinephrine, which are synthesized by tyrosine [11]. Epinephrine is linked to the regulation of body temperature, which is derived from the phenylalanine and tyrosine [11]. l-threonine is oxidized by threonine dehydrogenase to form glycine [12]. Glycine is transformed into serine by the action of serine hydroxymethyltransferase, and serine further forms cysteine [13]. Cysteine is dehydrogenated to form cystine, which in turn can be hydrogenated to cysteine. Cystine is a structural component of many tissues and hormones. Besides, cysteine is also extremely important for energy metabolism which could be oxidatived to deaminate pyruvate and synthesize taurine [9]. Taurine has a wide range of physiological and pharmacological effects and is important in order to regulating the body’s glucose and lipid metabolism. Pantothenic acid, vitamin B5, is a water-soluble vitamin required for life support which can form coenzyme A (CoA). CoA is mainly engaged in the breakdown of carbohydrates, fatty acid oxidation, amino acid decomposition, pyruvate degradation, and the tricarboxylic acid cycle. Pantothenic acid is critical to the metabolism and synthesis of carbohydrates, proteins, and fats and can also inhibit the metabolism of taurine [14]. Secretory epithelial cells of human and other animal prostate have unique citrate-related metabolic pathways regulated by testosterone and prolactin. This specialized hormone metabolic regulation is in charge of the production and secretion of a particularly high level of citric acid. Testosterone and prolactin are also engaged in the regulation of the gene of key regulatory enzymes of citrate production in prostate cells [15]. As the intermediate of the TCA cycle, malic acid and citric acid paticipate the metabolic pathways related to prostate [16]. Conclusively, TCA cycle is the hub of sugar, fat, and amino acid metabolism and most intermediates in the tricarboxylic acid cycle can be precisely or indirectly converted to various amino acids. Therefore, there are closely relationships among amino acid metabolism and energy metabolism (Figure 9). Besides, the results of Na^+^/K^+^-ATPase’s activities and respiratory research also verified energy variety in the procession of CNP. Studies have demonstrated that the flavonoids such as curcumin could inhibit TNF-α to exert anti-inflammatory effects [17]. The flavonoids in *T. patula* can also reduce the increasing of TNF-α levels caused by CNP. Here we supposed that the flavonoids could decrease the secretion of TNF-α and other inflammatory cytokines, which might associate with the structure of flavonoids. The structure of flavonoids usually contains phenolic hydroxyl and ketone carbonyl, which have antioxidant effects in the study of the structure-activity relationship [18]. We just found the flavonoids are the effective fraction against CNP and we still need to investigate the specific compound and its potential mechanisms in further study.

In conclusion, the potential anti-CNP mechanism of flavonoids and polysaccharides of *T. patula* might be through reducing the expression of inflammation factors, adjusting the levels of hormone and regulating the amino acid metabolism, energy metabolism and glucose and lipid metabolism. 

## 4. Materials and Methods

### 4.1. Materials and Reagents

*T. patula* was provided by Dalian Wuzhou Holy Herb Scientific and Technological Co. Ltd. (Dalian, China) and identified by Prof. Bing Wang in the Liaoning University of Traditional Chinese Medicine, the voucher specimens (No. 20160911) were deposited in the specimen herbarium, Liaoning University of Traditional Chinese Medicine. Acetonitrile, methanol and formic acid (HPLC grade) were acquired from Merck (Darmstadt, Germany). Milli-Q water purification system (Millipore, Bedford, MA, USA) was used for the purification of water and the preparation of samples and mobile phase. Estradiol benzoate was purchased from the Ningbo Second Hormone Factory (Ningbo, China). Levofloxacin hydrochloride was acquired from the Cisen Pharmaceutical Co., Ltd. (Jining, China). DHT and PSA ELISA kits were purchased from Shanghai Qiaodu Biotechnology Co. Ltd. (Shanghai, China). TNF-α ELISA kit, Na^+^/K^+^-ATPase assay kit and Total protein quantitative assay kit were all purchased from the Nanjing Jiancheng Biotechnology Co. Ltd. (Nanjing, China). 2-ketoglutaric acid, pyruvic acid, succinic acid, disodium fumarate, malic acid, stearic acid, 3-hydroxytyramine hydrochloride and mixed amino acid standards were purchased from Sigma (St. Louis., MO, USA). Betaine, allantoin, inosine, taurine, creatinine, l-carnitine, creatine, urea and citric acid were recruited from Aladdin (Shanghai, China). Sodium lactate, doxifluridine, d-(+)-pantothenic acid calcium salt and 6-hydroxypurine were purchased from the National Institutes for Food and Drug Control (Beijing, China). All the other chemicals employed in the experiments were commercial products of analytical grade. The preparation methods of each constituents are as follows, *T. patula* was extracted with distilled water and boiling to obtain water decoction. *T. patula* was extracted with distilled water and boiling to get the essential oil components with essential oil extractor. The resulting decoction was filtered. The residual was continued to extract with distilled water, and then, filtered. The filtrates and essential oil were collected respectively. The collected filtrates were concentrated, then adding alcohol to the concentrated solution to meet 70% alcohol for two times to provide the precipitate and supernatant constituents, respectively. The precipitate equal to POL and supernatant equal to FLA. The concrete preparation methods and administration solutions of different constituents of *T. patula* were obtained according to the reference [3].

### 4.2. Animals

Adult male SPF level Sprague-Dawley rats (weighing approximately 180–200 g) were offered by the Liaoning Changsheng Biotechnology Co. Ltd, Liaoning, China (License Key: SCXK (Liao) 20150001) and maintained under controlled condition (20 ± 2 °C, 40 ± 10% relative humidity and 12 hours light/dark cycle) with free access to standard food and water. Animal research was approved by the Animal Ethical and Welfare Committee of Liaoning University of Traditional Chinese Medicine and the experimental protocols were conducted according to the Guide for Care and Use of Laboratory Animals of Liaoning University of Traditional Chinese Medicine (131/2010).

After one week of acclimatization, the CNP model was induced by estradiol subcutaneously in castrated male rats according to the methods which established as Robinette described [19]. After the model was performed, the rats were randomly divided into 8 groups with 8 animals for each and intragastrically administrated with corresponding drugs for 9 days. SOG, MOG, WAD (1.8 g/kg/day), EOC (5.4 mg/kg/day), POL (254.3 mg/kg/day), FLA (207.5 mg/kg/day), TCM (540 mg/kg/day) and LEH (45 mg/kg/day) were intragastrically administrated with the corresponding drugs. And SOG and MOG were intragastrically administrated with 0.5% CMC-Na. The preparation methods of constituents of *T. patula* and the concentration of drugs for intragastrically administration was equated with the dose of human daily taking according to the reference [3]. 

### 4.3. Determination Eenergy Metabolic Parameters

After drugs administered on the 8th day 8:00 am, the rats in different groups were individually placed in the respiratory chambers of TSE phenoMaster/LabMaster (TSE phenoMaster/LabMaster, Bad Homburg, Germany) to monitor 24 h respiratory parameters in an isolated environment which temperature was controlled at 20 ± 2 °C. VO_2_, VCO_2_, H, EE and RER were measured by using the indirect calorimetry system.

### 4.4. Collection and Preparation of Biosamples

Urine samples were collected by metabolism cages at ambient temperature on the 9th of drug administration and centrifuged at 13,000 rpm at 4 °C for 10 min and stored frozen at −80 °C for metabolomic analysis.

### 4.5. Physiological Indexes

All rats were anesthetized after 24 h of the ending of the intragastric administration. Blood was drawn from the abdominal aorta. The serum was prepared and stored at −80 °C before estimation. Prostate and liver tissues were collected and stored at −80 °C. The left lateral lobe part of prostate were fixed in 4% paraformaldehyde solution for histological evaluation. Homogenate was obtained according to the ratio of tissue of right lateral lobe of prostate or liver and saline 1:9. The supernatant was harvested by centrifugation for 10 min at 3000 rpm. The levels of DHT and PSA in serum and TNF-α in prostate homogenate and Na^+^/K^+^-ATPase activity in prostate and liver homogenate were measured with correspondent measurement kit.

### 4.6. Metabolic Profiling Chromatography

Chromatography was performed using a Waters ACQUITYTM ultra performance liquid chromatography system (Waters Corporation, Milford, CT, USA) controlled with Masslynx (V4.1, Waters Corporation, Milford, CT, USA). An aliquot of 4 µL of the sample solution was injected onto an ACQUITY UPLC HSS T3 column (50 mm × 2.1 mm, 1.7 µm, Waters Corp, Milford, CT, USA) at 40 °C with a flow rate of 0.60 mL/min. The mobile phases were composed of 0.1% formic acid in acetonitrile (solvent A) and 0.1% formic acid in water (solvent B), the gradient was used as Supplementary Materials Table S5: Gradient elution program. The eluant was introduced to the mass spectrometer (Waters Corporation, Milford, CT, USA) directly and analyzed with positive/negative electrospray ion source (ESI). The quality control (QC) sample was used to optimize the condition of UPLC-MS, as it contained most information on whole urine samples. After every 10 samples injected, a pooled sample as the QC sample followed by a blank was injected in order to secure the stability and repeatability of the UPLC-MS systems.

### 4.7. Mass Spectrometry

Metabolic was analyzed and identified by Micromass Q-TOF micro^TM^ mass spectrometry and the optimal conditions of analysis were as follows. In positive ion mode, the capillary voltage was 3.0 kV, the sampling cone voltage was 45 V, desolvation gas temperature was 400 °C, desolvation gas flow was 800 L/h, the cone gas flow was 50 L/h. The data acquisition rate was set to 1 sec/scan with a 0.2 second interscan delay and the mass range was set at *m*/*z* 50–1200 using extended dynamic range. For accurate and repeatable mass acquisition, a lock-mass of leucine-enkephalin at a concentration of 50 fmol/µL was used via a lock spray interface at a flow rate of 60 µL/min monitoring for positive ion mode ([M + H] = 556.2771) and negative ion mode ([M − H] = 554.2615) to ensure accuracy during the MS analysis.

### 4.8. Data Processing

Testing and matching original peak through Markerlynx (SCN803) modules in Masslynx V4.1 workstation, and the peak intensity was normalized and denoised to extract data matrix consisting of metabolite retention time, *m*/*z* and corresponding peak intensities. Software specific parameter settings are shown in Supplementary Materials Table S6: Markerlynx Software parameter settings.

### 4.9. Multivariate Data Analysis

Data preprocessed analysis by using the extended statistics module in Masslynx V4.1 Workstations for compound statistics. All the data containing the retention time, peak intensity and exact mass were imported in the Masslynx V4.1 Workstations for multiple statistical analyses. Both principal component analysis (PCA) and partial least square discriminant analysis (PLS-DA) often could be taken, because of their ability to deal with highly multivariate, noisy, collinear and possibly incomplete data. PCA, unsupervised pattern recognition method, used to discern the presence of inherent similarities in spectral profiles initially. In the PCA plot, the longer distance the two samples’ scores are, the greater the difference between them.

### 4.10. Biomarkers Identification

The retention time, precise molecular mass and *m*/*z* data for the structural identification of biomarkers were issued by the UPLC-MS analysis platform of the Masslynx V4.1 Workstations. The variable importance in projection value (VIP value) was obtained by PLS-DA, and the metabolites with VIP > 1 were selected to measure the significance of each metabolite in separating model from controls by one-way ANOVA with SPSS 17.0. Metabolites with VIP >1 and *p* < 0.05 were identified as differential metabolites and compound validation to verify the accuracy and eliminate unreliable differential metabolites.

Targeted Metabolomics: potential markers were identified by matching the retention time and *m*/*z* of these differential metabolites with the theoretical *m*/*z* based on the elemental composition of the standard products through the Molecular Weight Calculator module in Masslynx.

Non-targeted metabolomics: as a result of the relevant information on compounds structure types, elemental composition, and other information in urine samples were unknown, the identification of differential metabolites in this study mainly included the following methods. (1) Determination of differential metabolites [M + H]^+^ or [M − H]^−^ ions, through the molecular ion peak to find the possible elemental composition of the metabolite; (2) Analysis of elemental composition by MS and MS/MS spectra to find the fragment ion peaks of metabolites, according to the structure which metabolites might exist and meet the fragmentation peaks of mass spectrometry characteristics to infer the possible structures of the different metabolites and verified by an online database such as the MS/MS Spectrum Match module in the METLIN database (http://metlin.scripps.edu/). (3) Some of the metabolites did not split into debris due to the complexity of urine samples, the structure of metabolites were reverse analyzed of metabolically related networks and inferred through the possible mechanisms of prostatitis and according to the metabolic correlation networks which were identified by targeted metabolomics databases such as KEGG (http://www.genome.jp/kegg/), HMDB (http://www.hmdb.org/). 

### 4.11. Metabolic Pathway Analysis 

Metabolic pathways were obtained by analyzing and enriching differential metabolites that had been screened by MetaboAnalyst 3.0. Potential markers identified were compared with the accurate mass charge ratio in some databases, including HMDB, KEGG and Metlin database to discover related pathways. Pathway impact-alter was used to determine the statistical significance of the pathways.

### 4.12. Statistical Analysis

The PCA was used to uncover undiscovered trends in the treated groups. Statistically significant differences in mean values were tested by using one-way ANOVA, and *p* < 0.05 was considered statistically significant. Prior to multivariate analysis, the resultant data matrices from the two analytical techniques were mean-centered and pareto-scaled.

### 4.13. Network Pharmacology Predict Pathway

In the early stages, our laboratory was separated and identified the compound in *T. patula* and found that flavonoids is the main active component. Here, to certify the result of metabolism, the network-pharmacology was operating to illustrate the predictive action mechanism. The information of the active compound in *T. patula* was shown in Supplementary Materials Table S7: Structures of active compounds. Prediction of pathways that may be related to *T. patula* anti-prostatitis by screening out the common target genes of *T. patula* active compounds and prostatitis.

Potential targets of active compounds were predicted by SwissTargetPrediction server (http://www.swisstargetprediction.ch/) and stinted homo sapiens as organism. According to the CTD (https://ctdbase.org/) and Genecard (https://www.genecards.org/) databases to find relevant target genes of prostatitis, due to the large number of related genes and the complexed network, so selected gene with relevance score >30 to predict. The network was constructed using Cytoscape 3.7.1 software [20] to obtain the active compounds of *T. patula*—target map and prostatitis disease—target map. The common targets gene of *T. patula* active compounds and prostatitis was screened out by the merge function of Cytoscape, and the *T. patula* active compounds–target–prostatitis disease network map was constructed. Then the analysis of gene function was performed on the merge gene utilizing FunRich software [21,22]. The DAVID database (https://david.ncifcrf.gov/tools.jsp) was combined with KOPAS 3.0 (http://kobas.cbi.pku.edu.cn/anno_iden.php) to enrich the KEGG and GO pathways in the gene pathway. After the pathway enrichment, the broad pathways were excluded and the pathway information was obtained. Then Omicshare cloud platform (http://www.omicshare.com/forum/) was used to map the enriched pathways.

## Figures and Tables

**Table 1:** Differential Metabolite Pathway Analysis Results.

Compound ID	Metabolite	Molecular Formula	Mode	Ret. Time	*M*/*Z*	Trend
HMDB0000158	**Δ**l-tyrosine	C_9_H_11_NO_3_	ESI -	1.3987	180.0632	-
HMDB0006236	Phenylacetaldehyde	C_8_H_8_O	ESI +	1.5029	121.0663	-
HMDB0000205	Phenylpyruvic acid	C_9_H_8_O_3_	ESI -	3.5141	163.0386	-
HMDB0000159	**Δ**l-phenylalanine	C_9_H_11_NO_2_	ESI +	2.1193	120.0800	-
HMDB0003426	Pantetheine	C_11_H_22_N_2_O_4_S	ESI +	2.5712	279.1378	-
HMDB0000574	l-Cysteine	C_3_H_7_NO_2_S	ESI +	4.8468	122.0283	-
HMDB0000210	Pantothenic Acid	C_9_H_17_NO_5_	ESI -	2.3662	218.1016	-
HMDB0000300	Uracil	C_7_H_10_N_2_OS	ESI +	1.5955	113.0465	+
HMDB0000929	Tryptophan	C_11_H_12_N_2_O_2_	ESI +	2.9052	205.1002	+
HMDB0000259	Serotonin	C_10_H_12_N_2_O	ESI +	1.8983	177.1051	-
HMDB0000684	l-Kynurenine	C_10_H_12_N_2_O_3_	ESI +	2.3458	191.0838	-
HMDB0000734	Indoleacrylic acid	C_11_H_9_NO_2_	ESI -	6.86	186.0561	+
HMDB0000978	4-(2-Aminophenyl)-2,4-dioxobutanoic acid	C_10_H_9_NO_4_	ESI +	3.434	208.0615	-
HMDB0000197	3-Indoleacetic Acid	C_10_H_9_NO_2_	ESI -	6.1042	174.0572	-
HMDB0001644	d-Xylulose	C_5_H_10_O_5_	ESI -	1.6288	151.0607	-
HMDB0000127	d-Glucuronic acid	C_6_H_10_O_7_	ESI -	3.6827	193.0474	+
HMDB0000177	**Δ**l-histidine	C_6_H_9_N_3_O_2_	ESI +	0.6353	156.0459	-
HMDB0062781	2-oxoglutarate	C_5_H_6_O_5_	ESI -	0.8023	145.0105	+
HMDB0000156	**Δ**l-Malic acid	C_4_H_6_O_5_	ESI -	0.7309	133.0072	+
HMDB0000094	**Δ** Citrate	C_6_H_8_O_7_	ESI +	0.9318	193.0375	+
HMDB0000068	Epinephrine	C_6_H_7_N_5_O	ESI +	2.3582	184.0994	-
HMDB0000073	**Δ** Dopamine	C_8_H_11_NO_2_	ESI +	0.8783	154.0985	+
HMDB0002052	Maleylacetoacetic acid	C_8_H_8_O_6_	ESI +	2.8867	201.042	-
HMDB0000251	**Δ** Taurine	C_2_H_7_NO_3_S	ESI -	0.6159	124.0016	-
HMDB0000167	**Δ**l-threonine	C_4_H_9_NO_3_	ESI +	0.7366	120.0690	+
HMDB0000123	**Δ** Glycine	C_2_H_5_NO_2_	ESI +	4.2937	76.0406	-
HMDB0000289	Uric acid	C_5_H_4_N_4_O_3_	ESI +	1.0763	169.0381	-
HMDB0000034	Adenine	C_6_H_7_N_5_	ESI +	0.9096	136.0654	-

Notes: **Δ** represents target metabolomics differential metabolites, and the rest are untargeted metabolomics differential metabolites. - indicates that MOG has a decreasing trend compared with SOG, + indicates that MOG showed an upward trend compared with SOG.

**Table 2:** Metabolyst Approach Analysis Results Based on KEGG.

No.	Pathway Name	Match Status	P	Impact
1	**Δ** Phenylalanine, tyrosine and tryptophan biosynthesis	3/4	2.52 × 10^−5^	1
2	**Δ** Phenylalanine metabolism	4/9	1.29 × 10^−5^	0.77778
3	**Δ** Taurine and hypotaurine metabolism	2/8	0.0093149	0.42857
4	Ascorbate and aldarate metabolism	1/9	0.16098	0.4
5	**Δ** Tryptophan metabolism	5/41	8.37 × 10^−4^	0.35955
6	**Δ** Tyrosine metabolism	4/42	0.0074067	0.32959
7	**Δ** Glyoxylate and dicarboxylate metabolism	2/16	0.036307	0.2963
8	**Δ** Glycine, serine and threonine metabolism	3/32	0.021783	0.29197
9	Histidine metabolism	1/15	0.25411	0.24194
10	**Δ** Citrate cycle (TCA cycle)	3/20	0.0058413	0.16675
11	Cysteine and methionine metabolism	1/28	0.42297	0.12829
12	Pantothenate and CoA biosynthesis	4/15	1.29 × 10^−4^	0.08163
13	Alanine, aspartate and glutamate metabolism	1/24	0.37538	0.06329
14	Primary bile acid biosynthesis	2/46	0.22107	0.05952
15	Pyrimidine metabolism	1/41	0.5547	0.04182
16	Purine metabolism	2/68	0.38017	0.02549
17	Glutathione metabolism	2/26	0.087435	0.00955
18	Ubiquinone and other terpenoid-quinone biosynthesis	1/3	0.056709	0
19	Thiamine metabolism	1/7	0.12752	0
20	Starch and sucrose metabolism	1/23	0.36291	0
21	Pyruvate metabolism	1/22	0.35019	0
22	Porphyrin and chlorophyll metabolism	1/27	0.41141	0
23	Pentose and glucuronate interconversions	2/14	0.028192	0
24	Nitrogen metabolism	2/9	0.011835	0
25	Methane metabolism	1/9	0.16098	0
26	Inositol phosphate metabolism	1/26	0.39963	0
27	d-glutamine and d-glutamate metabolism	1/5	0.09278	0
28	Cyanoamino acid metabolism	1/6	0.11031	0
29	Butanoate metabolism	1/20	0.32403	0
30	beta-Alanine metabolism	1/19	0.31057	0
31	Aminoacyl-tRNA biosynthesis	7/67	1.72 × 10^−4^	0

Notes: **Δ** represents pathway satisfied with the condition of both *p* < 0.05 and impact > 0.1

## Supplementary Materials

The following are available online at https://www.mdpi.com/1420-3049/24/12/2266/s1, Figure S1: HPLC fingerprint of water decoction (a), supernatant (b) and essential oil (c) fractions of *T. patula*., Figure S2: TIC chromatograms on positive ion mode, Figure S3: TIC chromatograms on negative ion mode, Figure S4 Histological analysis of prostate, Table S1: Potential targets of active compounds, Table S2: Relevant target genes of prostatitis, Table S3: The information of merging gene, Table S4: Details of enriched pathways, Table S5: Gradient elution program, Table S6: Markerlynx Software parameter settings, Table S7: Structures of active compounds.

## Abbreviations

CNP	Chronic nonbacterial prostatitis
PSA	prostate-specific antigen
TNF-α	tumor necrosis factor-α
DHT	dihydrotestosterone
SOG	Sham-operated group
MOG	Chronic nonbacterial prostatitis model group
WAD	Water decoction treatment group
EOC	Essential oil treatment group
POL	Polysaccharide treatment group
FLA	Flavonoids treatment group
TCM	Pule’an tablet treatment group
LEH	Levofloxacin treatment group
VO_2_	O_2_ consumption
VCO_2_	CO_2_ production
H	Heat production
EE	Energy expenditure
RER	Respiratory exchange ratio
ESI	electrospray ion source
QC	The quality control
PCA	principal component analysis
PLS-DA	partial least square discriminant analysis
