# Supplementary material for: Mechanism Investigation of Tagetes patula L. against Chronic Nonbacterial Prostatitis by Metabolomics and Network Pharmacology
Source: Molecules. 2019 Jun 18;24(12):2266. doi: 10.3390/molecules24122266 (PMC6630244; doi:10.3390/molecules24122266)
Supplement: Supplementary file 1 [file molecules-24-02266-s001.pdf]

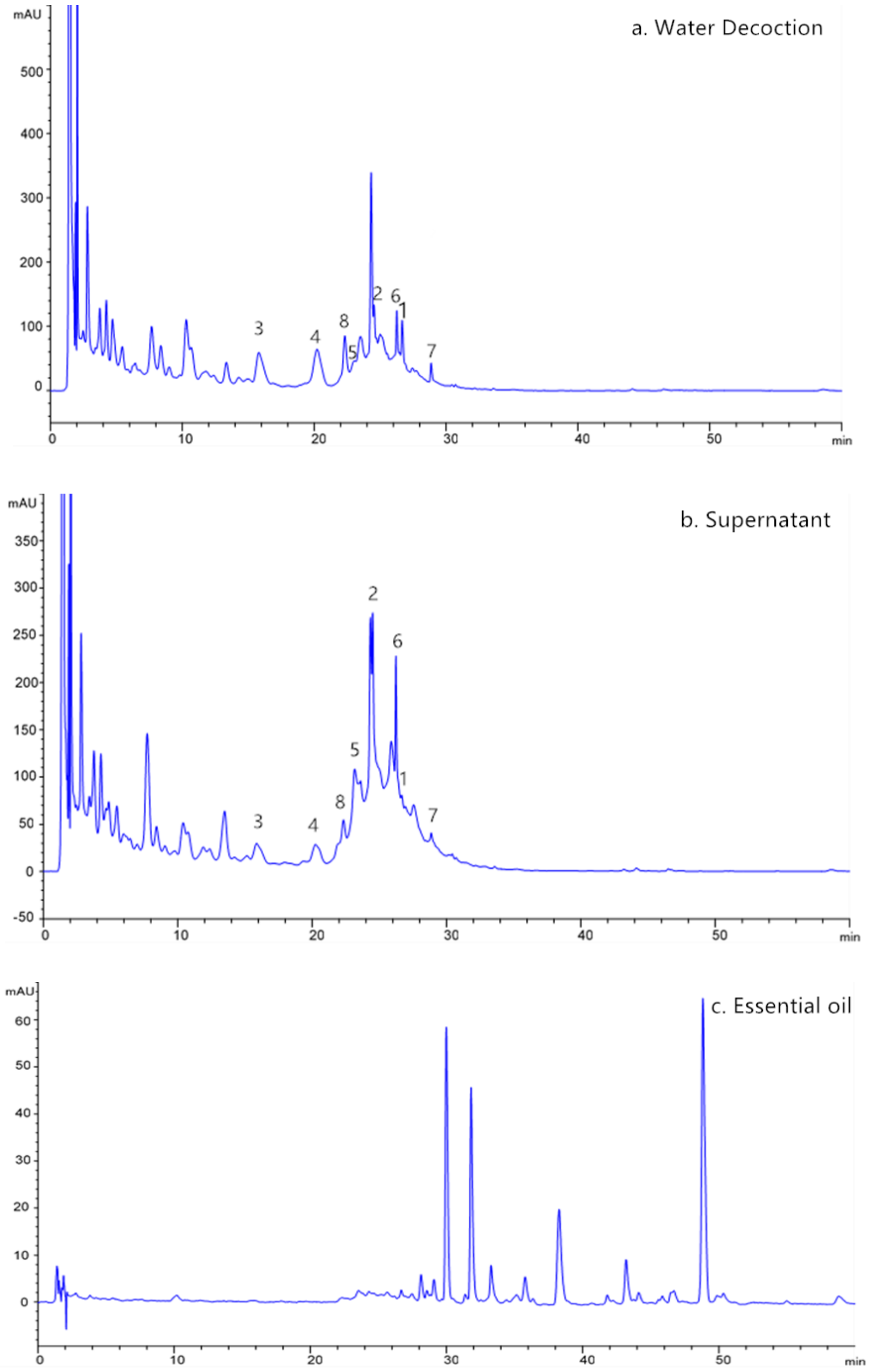

Figure S1 HPLC fingerprint of water decoction(a), supernatant(b) and essential oil(c) fractions of *T. patula*.

Notes: (1) patuletin, (2) quercetin-7-*O*-*α*-L-rhamnosepyranoside, (3) quercetin-3-*O*-*α*-L-arabinopyranoside, (4) kaempferol-3-*O*-*β*-D-glucopyranoside, (5) kaempferol-3-*O*-*β*-D-xylopyranoside, (6) kaempferol-3-*O*-*α*-L-rhamnosepyranoside, (7) kaempferol, (8) kaempferol-3-*O*-*α*-L-arabonipyranoside.

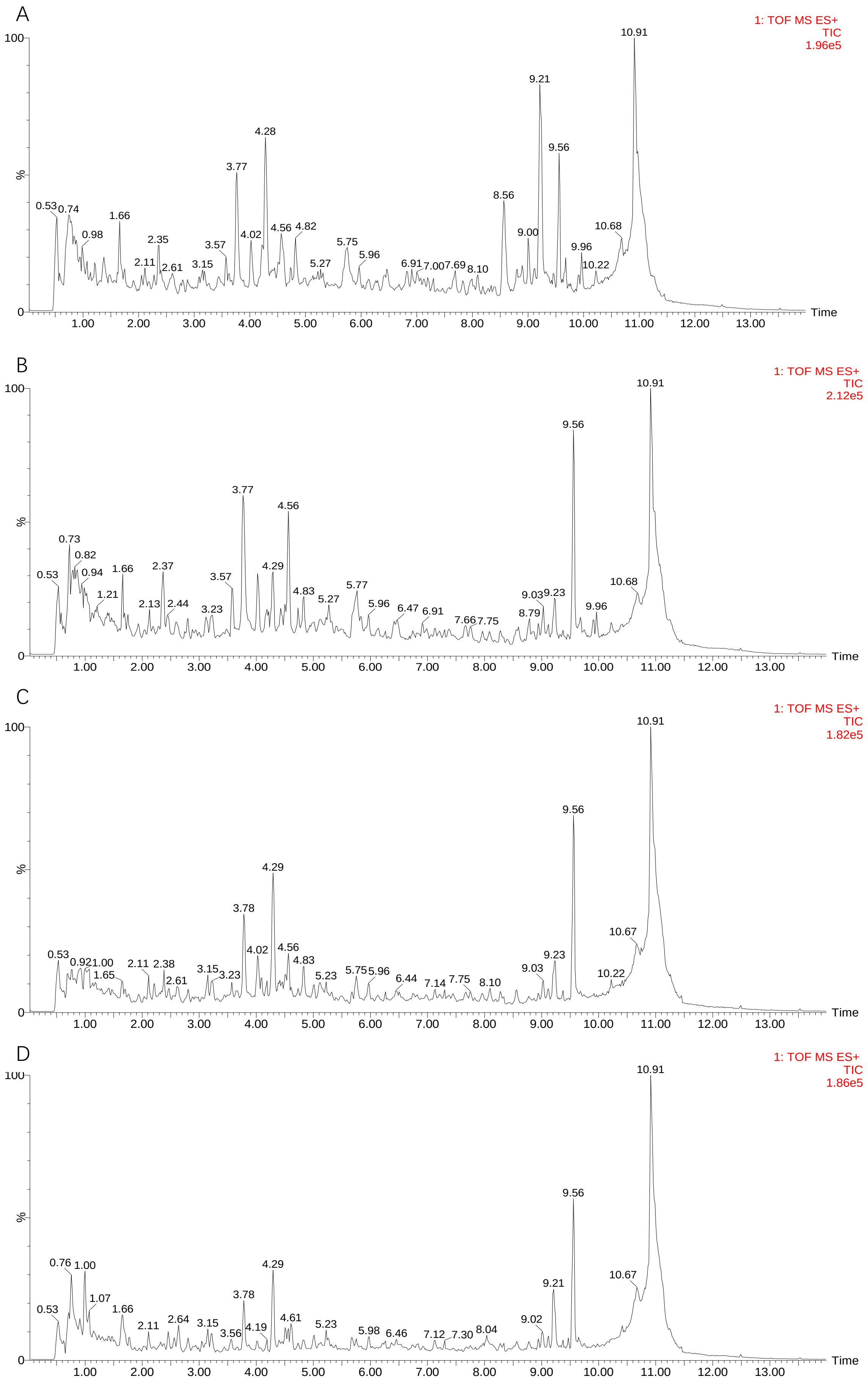
A
1: TOF MS ES+
TIC
1.96e5
100
%
0
Time
10.91
9.21
4.28
9.56
3.77
8.56
0.53
0.74
1.66
0.98
4.02
4.56
4.82
2.35
3.57
9.00
10.68
5.75
9.96
5.96
2.11
3.15
5.27
6.91
7.00
7.69
10.22
2.61
8.10
1.00
2.00
3.00
4.00
5.00
6.00
7.00
8.00
9.00
10.00
11.00
12.00
13.00
B
1: TOF MS ES+
TIC
2.12e5
10.91
9.56
3.77
4.56
0.73
0.82
0.94
1.66
2.37
4.29
3.57
0.53
1.21
4.83
5.77
9.03
9.23
10.68
2.13
2.44
5.27
5.96
3.23
6.47
6.91
9.96
8.79
7.66
7.75
C
1: TOF MS ES+
TIC
1.82e5
10.91
9.56
4.29
3.78
10.67
0.53
9.23
4.02
4.56
0.92
1.00
2.11
2.38
4.83
1.65
3.15
3.23
5.75
5.96
9.03
10.22
2.61
5.23
6.44
7.14
7.75
8.10
D
1: TOF MS ES+
TIC
1.86e5
10.91
9.56
0.76
1.00
4.29
10.67
9.21
3.78
1.07
0.53
1.66
2.64
4.61
2.11
3.15
4.19
5.23
3.56
5.98
6.46
7.12
7.30
8.04
9.02

E

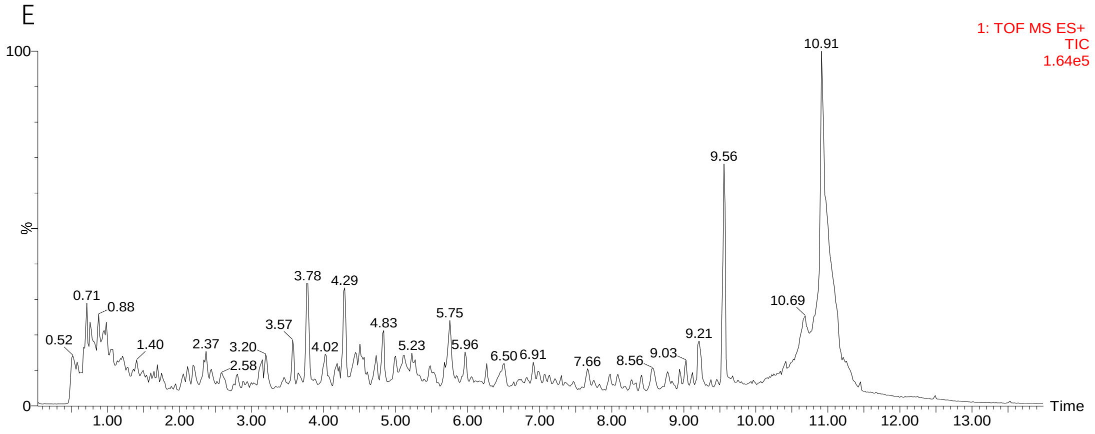
1: TOF MS ES+
TIC
1.64e5
100
%
0
0.52
0.71
0.88
1.40
2.37
2.58
3.20
3.57
3.78
4.02
4.29
4.83
5.23
5.75
5.96
6.50
6.91
7.66
8.56
9.03
9.21
9.56
10.69
10.91
Time
1.00
2.00
3.00
4.00
5.00
6.00
7.00
8.00
9.00
10.00
11.00
12.00
13.00

F

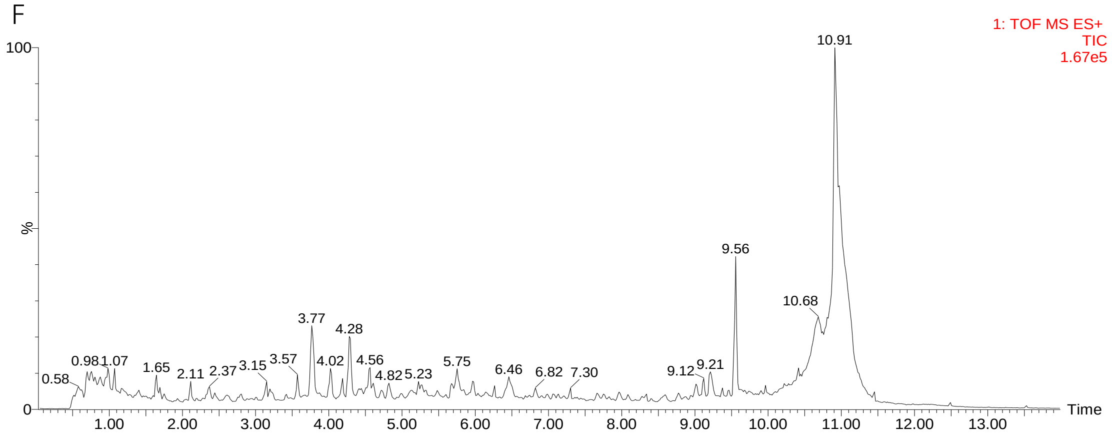
1: TOF MS ES+
TIC
1.67e5
100
%
0
0.58
0.98
1.07
1.65
2.11
2.37
3.15
3.57
3.77
4.02
4.28
4.56
4.82
5.23
5.75
6.46
6.82
7.30
9.12
9.21
9.56
10.68
10.91
Time
1.00
2.00
3.00
4.00
5.00
6.00
7.00
8.00
9.00
10.00
11.00
12.00
13.00

G

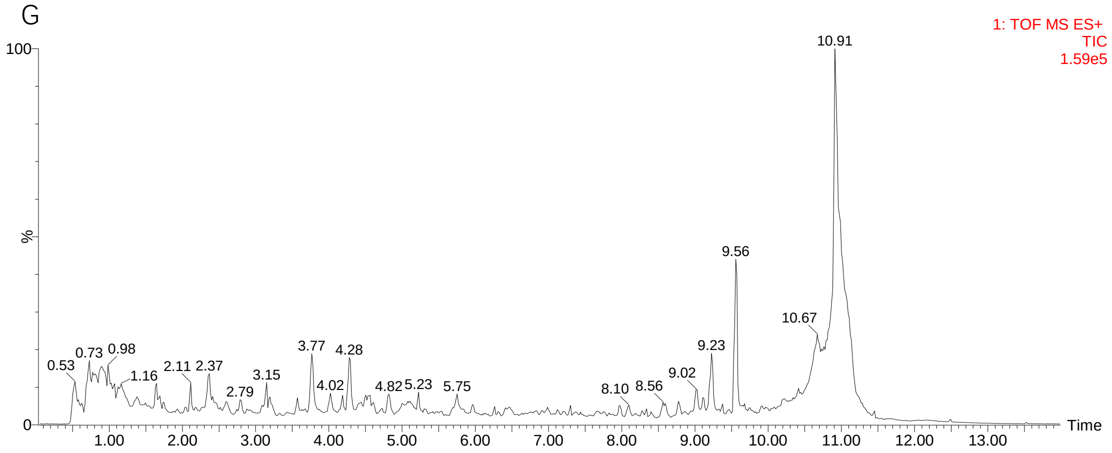
1: TOF MS ES+
TIC
1.59e5
100
%
0
0.53
0.73
0.98
1.16
2.11
2.37
2.79
3.15
3.77
4.02
4.28
4.82
5.23
5.75
8.10
8.56
9.02
9.23
9.56
10.67
10.91
Time
1.00
2.00
3.00
4.00
5.00
6.00
7.00
8.00
9.00
10.00
11.00
12.00
13.00

H

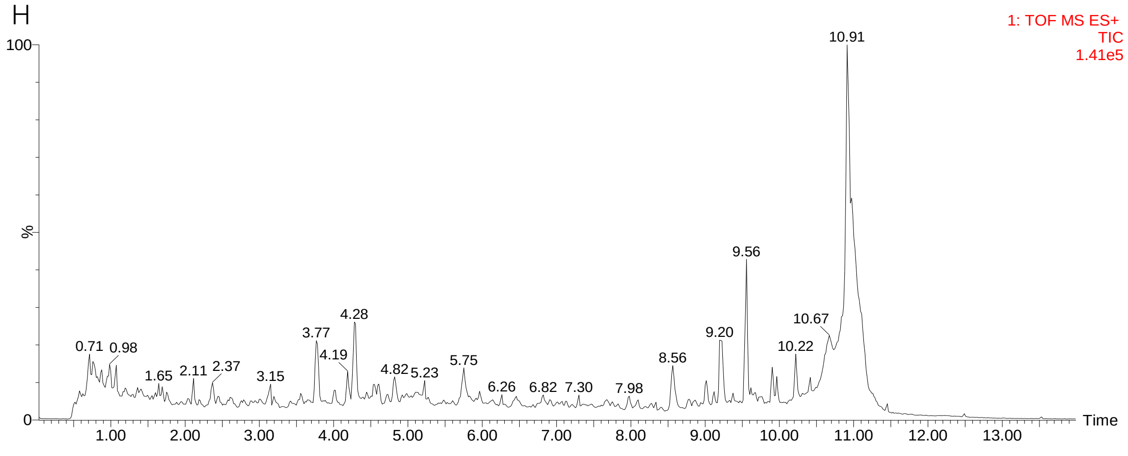
1: TOF MS ES+
TIC
1.41e5
100
%
0
0.71
0.98
1.65
2.11
2.37
3.15
3.77
4.19
4.28
4.82
5.23
5.75
6.26
6.82
7.30
7.98
8.56
9.20
9.56
10.22
10.67
10.91
Time
1.00
2.00
3.00
4.00
5.00
6.00
7.00
8.00
9.00
10.00
11.00
12.00
13.00

Figure S2 TIC chromatograms on positive ion mode

Notes:

A. Sham-operated Group

B. CNP Model Group

C. Levofloxacin Treatment Group

D. Pule' an Tablet Treatment Group

E. Water Decoction Treatment Group

F. Essential Oil Treatment Group

G. Polysaccharide Treatment Group

H. Supernatant Treatment Group

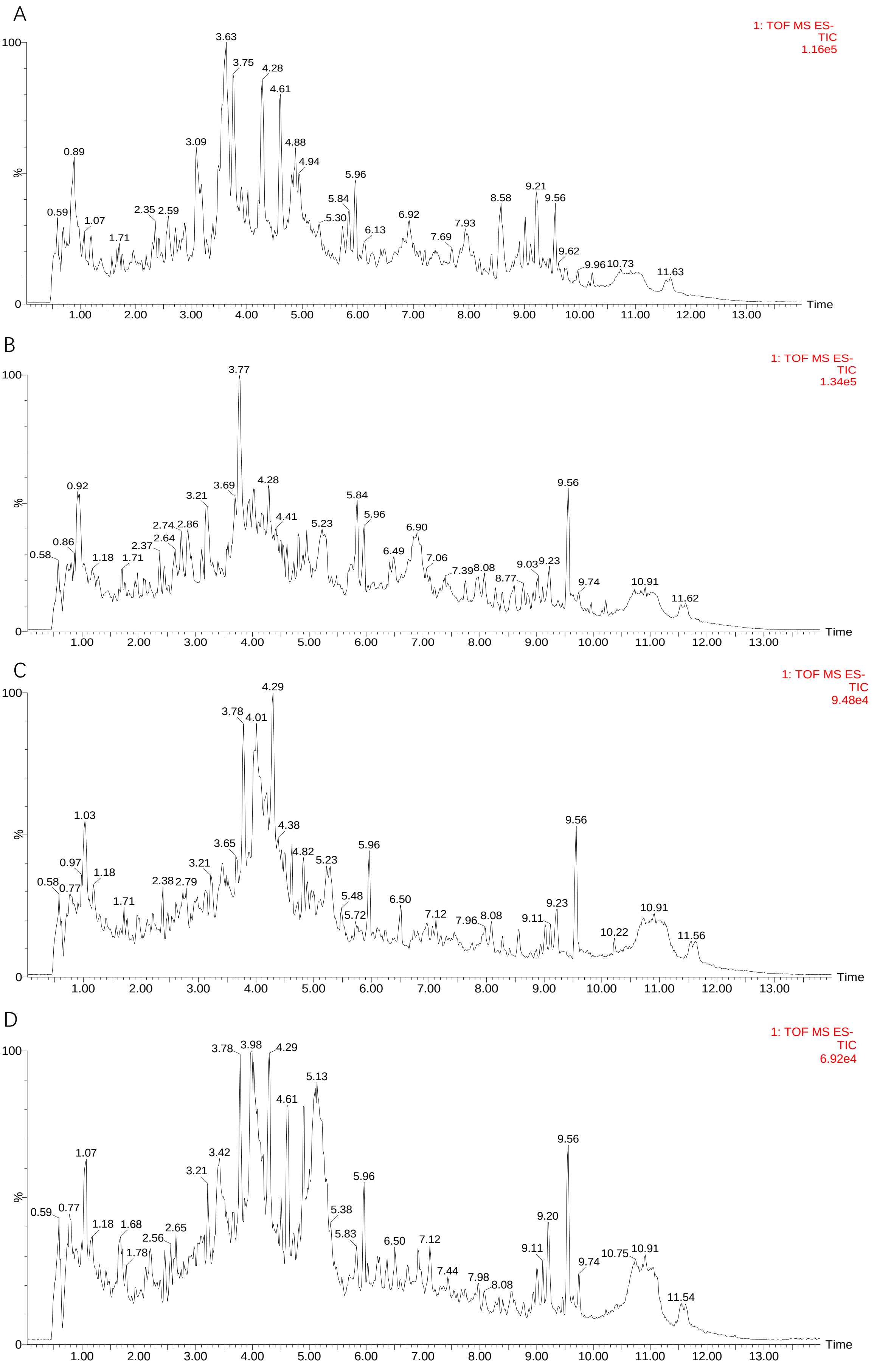

A
1: TOF MS ES-
TIC
1.16e5
B
1: TOF MS ES-
TIC
1.34e5
C
1: TOF MS ES-
TIC
9.48e4
D
1: TOF MS ES-
TIC
6.92e4
%
Time

E

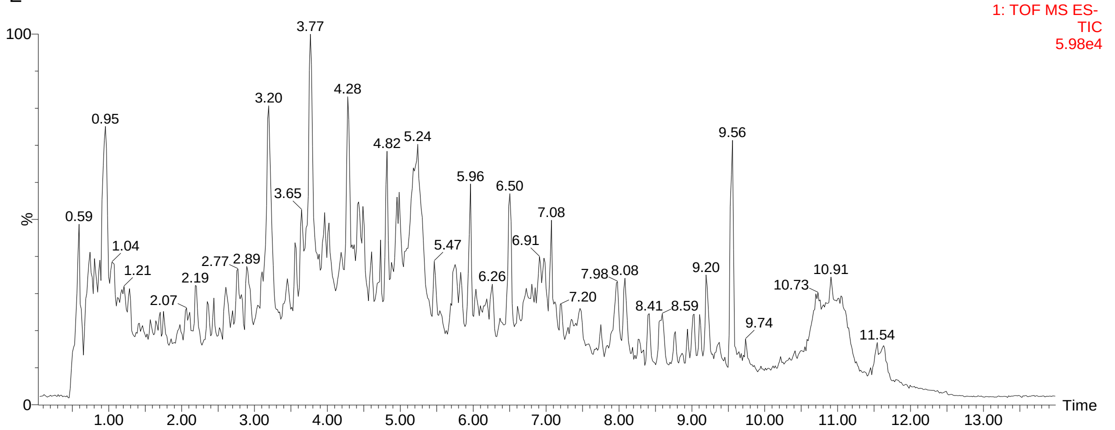
1: TOF MS ES-
TIC
5.98e4
100
%
0
3.77
3.20
4.28
0.95
4.82
5.24
9.56
3.65
5.96
6.50
0.59
7.08
1.04
6.91
5.47
1.21
2.77
2.89
2.19
6.26
7.98
8.08
9.20
10.91
10.73
2.07
7.20
8.41
8.59
9.74
11.54
Time
1.00
2.00
3.00
4.00
5.00
6.00
7.00
8.00
9.00
10.00
11.00
12.00
13.00

F

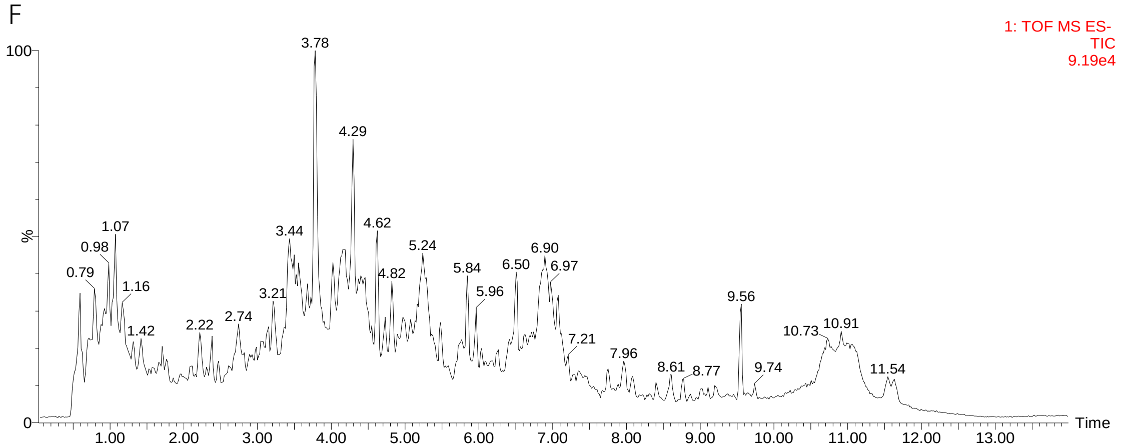
1: TOF MS ES-
TIC
9.19e4
100
%
0
3.78
4.29
1.07
3.44
4.62
0.98
5.24
6.90
0.79
4.82
5.84
6.50
6.97
1.16
5.96
3.21
9.56
2.74
10.91
2.22
10.73
1.42
7.21
7.96
8.61
8.77
9.74
11.54
Time
1.00
2.00
3.00
4.00
5.00
6.00
7.00
8.00
9.00
10.00
11.00
12.00
13.00

G

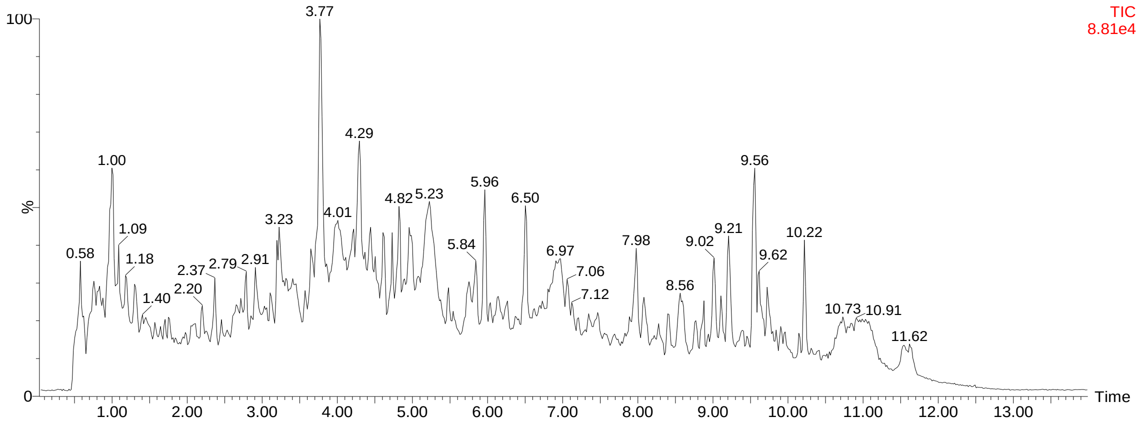
1: TOF MS ES-
TIC
8.81e4
100
%
0
3.77
4.29
1.00
9.56
5.96
4.82
5.23
6.50
3.23
4.01
1.09
9.21
10.22
5.84
9.02
7.98
0.58
6.97
9.62
1.18
2.37
2.79
2.91
7.06
2.20
8.56
1.40
7.12
10.73
10.91
11.62
Time
1.00
2.00
3.00
4.00
5.00
6.00
7.00
8.00
9.00
10.00
11.00
12.00
13.00

H

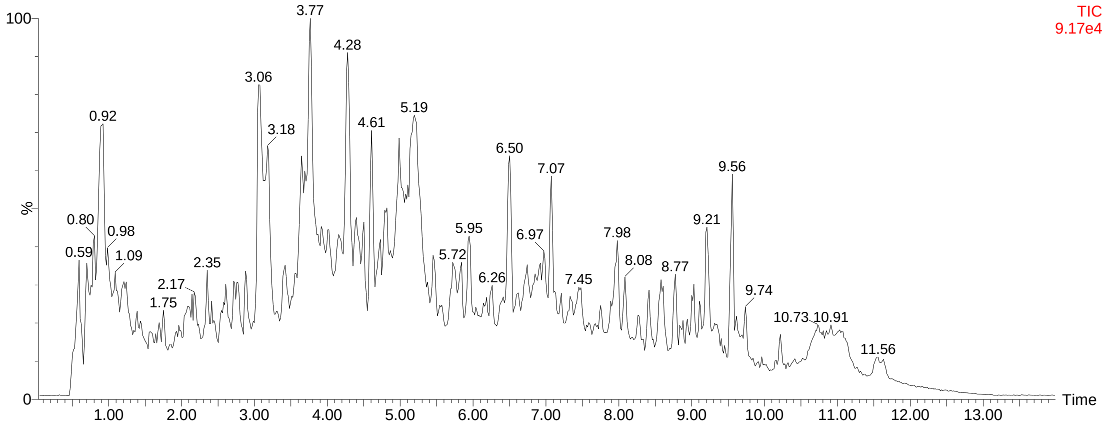
1: TOF MS ES-
TIC
9.17e4
100
%
0
3.77
4.28
3.06
0.92
5.19
3.18
4.61
6.50
7.07
9.56
0.80
0.98
5.95
6.97
7.98
9.21
0.59
1.09
5.72
8.08
8.77
2.35
6.26
7.45
2.17
1.75
9.74
10.73
10.91
11.56
Time
1.00
2.00
3.00
4.00
5.00
6.00
7.00
8.00
9.00
10.00
11.00
12.00
13.00

Figure S3 TIC chromatograms on negative ion mode

Notes:

A. Sham-operated Group

B. CNP Model Group

C. Levofloxacin Treatment Group

D. Pule' an Tablet Treatment Group

E. Water Decoction Treatment Group

F. Essential Oil Tratment Group

G. Polysaccharid Treatment Group

H. Supernatant reatment Group

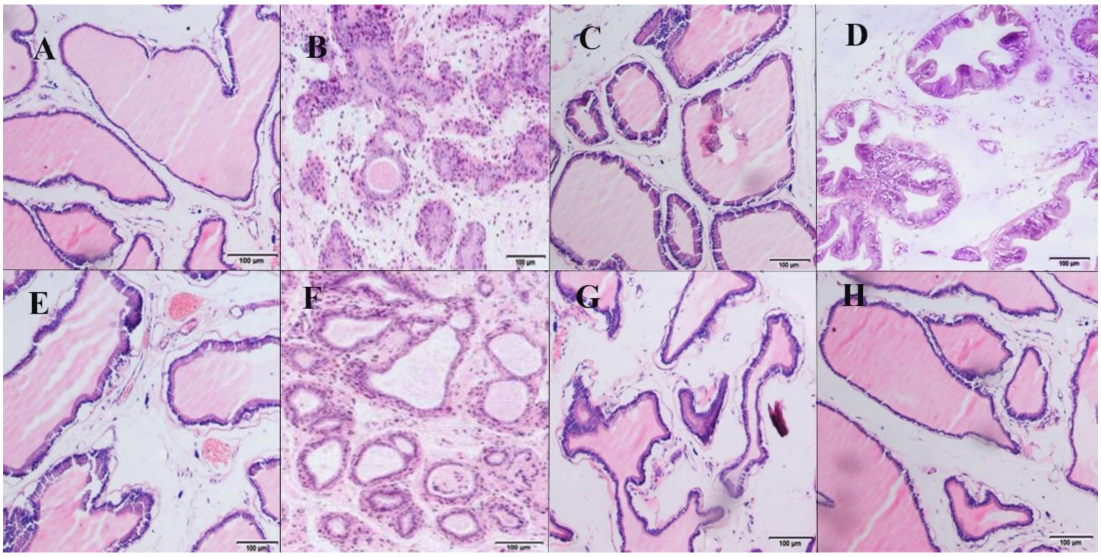

Figure S4 Histological analysis of prostate

Notes:

A. Sham-operated Group

B. CNP Model Group

C. Levofloxacin Treatment Group

D. Pule’ an Tablet Treatment Group

E. Water Decoction Treatment Group

F. Essential Oil Tratment Group

G. Polysaccharid Treatment Group

H. Supernatant reatment Group

Table S1 Potential targets of active compounds

Compound	Target	Uniprot ID	Gene Code	Probability
KQCR1	Tyrosyl-DNA phosphodiesterase 1	Q9NUW8	TDP1	0.86
	Muscleblind-like protein 1	Q9NR56	MBNL1	0.67
	Muscleblind-like protein 2	Q5VZF2	MBNL2	0.67
	Muscleblind-like protein 3	Q9NUK0	MBNL3	0.67
	Adenosine receptor A1	P30542	ADORA1	0.6
	Arachidonate 5-lipoxygenase	P09917	ALOX5	0.56
	Arachidonate 15-lipoxygenase	P16050	ALOX15	0.56
	Arachidonate 12-lipoxygenase	P18054	ALOX12	0.56
	Arachidonate 15-lipoxygenase B	O15296	ALOX15B	0.56
	Arachidonate 12-lipoxygenase	O75342	ALOX12B	0.56
	Epidermis-type lipoxygenase 3	Q9BYJ1	ALOXE3	0.56
	Multidrug resistance-associated protein 1	P33527	ABCC1	0.53
	Canalicular multispecific organic anion transporter 2	O15438	ABCC3	0.53
	Canalicular multispecific organic anion transporter 1	Q92887	ABCC2	0.53
	Dual specificity tyrosine-phosphorylation-regulated kinase 1A	Q13627	DYRK1A	0.51
KQCR2	Aldose reductase	P15121	AKR1B1	0.97
	Aldo-keto reductase family 1 member B15	C9JRZ8	AKR1B15	0.97
	Aldo-keto reductase family 1 member B10	O60218	AKR1B10	0.97
	Alcohol dehydrogenase [NADP(+)]	P14550	AKR1A1	0.96
	Dual specificity tyrosine-phosphorylation-regulated kinase 1A	Q13627	DYRK1A	0.96
	15-anhydro-D-fructose reductase	Q96JD6	AKR1E2	0.96
	Tyrosyl-DNA phosphodiesterase 1	Q9NUW8	TDP1	0.96
	Alpha-2A adrenergic receptor	P08913	ADRA2A	0.95
	Alpha-2C adrenergic receptor	P18825	ADRA2C	0.95
	NADPH oxidase 4	Q9NPH5	NOX4	0.95
	Alpha-2B adrenergic receptor	P18089	ADRA2B	0.95
	Ribosyldihydronicotinamide dehydrogenase [quinone]	P16083	NQO2	0.91
	NAD(P)H dehydrogenase [quinone] 1	P15559	NQO1	0.91
	Prostaglandin G/H synthase 1	P23219	PTGS1	0.87
	Prostaglandin G/H synthase 2	P35354	PTGS2	0.87
KQCR5	Aldose reductase	P15121	AKR1B1	0.98
	Aldo-keto reductase family 1 member B15	C9JRZ8	AKR1B15	0.98
	Aldo-keto reductase family 1 member B10	O60218	AKR1B10	0.98
	Dual specificity tyrosine-phosphorylation-regulated kinase 1A	Q13627	DYRK1A	0.98
	Alcohol dehydrogenase [NADP(+)]	P14550	AKR1A1	0.96
	5-anhydro-D-fructose reductase	Q96JD6	AKR1E2	0.96
	Tyrosyl-DNA phosphodiesterase 1	Q9NUW8	TDP1	0.94

	Ribosyldihydronicotinamide dehydrogenase [quinone]	P16083	NQO2	0.94
	NAD(P)H dehydrogenase [quinone] 1	P15559	NQO1	0.94
	Alpha-2A adrenergic receptor	P08913	ADRA2A	0.93
	Alpha-2C adrenergic receptor	P18825	ADRA2C	0.93
	NADPH oxidase 4	Q9NPH5	NOX4	0.93
	Alpha-2B adrenergic receptor	P18089	ADRA2B	0.93
	Muscleblind-like protein 1	Q9NR56	MBNL1	0.89
	Muscleblind-like protein 2	Q5VZF2	MBNL2	0.89
KQCR6	Aldose reductase	P15121	AKR1B1	0.94
	Aldo-keto reductase family 1 member B15	C9JRZ8	AKR1B15	0.94
	Aldo-keto reductase family 1 member B10	O60218	AKR1B10	0.94
	Dual specificity tyrosine-phosphorylation-regulated kinase 1A	Q13627	DYRK1A	0.94
	Alcohol dehydrogenase [NADP(+)]	P14550	AKR1A1	0.93
	5-anhydro-D-fructose reductase	Q96JD6	AKR1E2	0.93
	Ribosyldihydronicotinamide dehydrogenase [quinone]	P16083	NQO2	0.92
	NAD(P)H dehydrogenase [quinone] 1	P15559	NQO1	0.92
	Tyrosyl-DNA phosphodiesterase 1	Q9NUW8	TDP1	0.9
	Alpha-2A adrenergic receptor	P08913	ADRA2A	0.89
	Alpha-2C adrenergic receptor	P18825	ADRA2C	0.89
	NADPH oxidase 4	Q9NPH5	NOX4	0.89
	Alpha-2B adrenergic receptor	P18089	ADRA2B	0.89
	Prostaglandin G/H synthase 1	P23219	PTGS1	0.86
	Prostaglandin G/H synthase 2	P35354	PTGS2	0.86
KQCR9	Adenosine receptor A1	P30542	ADORA1	0.84
	Aldose reductase	P15121	AKR1B1	0.83
	Aldo-keto reductase family 1 member B15	C9JRZ8	AKR1B15	0.83
	Aldo-keto reductase family 1 member B10	O60218	AKR1B10	0.83
	Tyrosyl-DNA phosphodiesterase 1	Q9NUW8	TDP1	0.82
	Dual specificity tyrosine-phosphorylation-regulated kinase 1A	Q13627	DYRK1A	0.69
	Alpha-2A adrenergic receptor	P08913	ADRA2A	0.65
	Alpha-2C adrenergic receptor	P18825	ADRA2C	0.65
	Alpha-2B adrenergic receptor	P18089	ADRA2B	0.65
	Alcohol dehydrogenase [NADP(+)]	P14550	AKR1A1	0.65
	5-anhydro-D-fructose reductase	Q96JD6	AKR1E2	0.65
	Muscleblind-like protein 1	Q9NR56	MBNL1	0.65
	Muscleblind-like protein 2	Q5VZF2	MBNL2	0.65
	Muscleblind-like protein 3	Q9NUK0	MBNL3	0.65
	NADPH oxidase 4	Q9NPH5	NOX4	0.6
KQCR11	Cytochrome P450 1A2	P05177	CYP1A2	1
	Multidrug resistance protein 1	P08183	ABCB1	1

	Arachidonate 5-lipoxygenase	P09917	ALOX5	1
	Estradiol 17-beta-dehydrogenase 1	P14061	HSD17B1	1
	Aldose reductase	P15121	AKR1B1	1
	Arachidonate 15-lipoxygenase	P16050	ALOX15	1
	Arachidonate 12-lipoxygenase	P18054	ALOX12	1
	Multidrug resistance-associated protein 1	P33527	ABCC1	1
	Aryl hydrocarbon receptor	P35869	AHR	1
	Estradiol 17-beta-dehydrogenase 2	P37059	HSD17B2	1
	Xanthine dehydrogenase/oxidase	P47989	XDH	1
	Dual specificity tyrosine-phosphorylation-regulated kinase 1A	Q13627	DYRK1A	1
	Cytochrome P450 1B1	Q16678	CYP1B1	1
	NADPH oxidase 4	Q9NPH5	NOX4	1
	Tyrosyl-DNA phosphodiesterase 1	Q9NUW8	TDP1	1
KQCR12	Aldose reductase	P15121	AKR1B1	0.94
	Aldo-keto reductase family 1 member B15	C9JRZ8	AKR1B15	0.94
	Aldo-keto reductase family 1 member B10	O60218	AKR1B10	0.94
	Dual specificity tyrosine-phosphorylation-regulated kinase 1A	Q13627	DYRK1A	0.94
	Alcohol dehydrogenase [NADP(+)]	P14550	AKR1A1	0.93
	5-anhydro-D-fructose reductase	Q96JD6	AKR1E2	0.93
	Ribosyldihydronicotinamide dehydrogenase [quinone]	P16083	NQO2	0.92
	NAD(P)H dehydrogenase [quinone] 1	P15559	NQO1	0.92
	Tyrosyl-DNA phosphodiesterase 1	Q9NUW8	TDP1	0.9
	Alpha-2A adrenergic receptor	P08913	ADRA2A	0.89
	Alpha-2C adrenergic receptor	P18825	ADRA2C	0.89
	NADPH oxidase 4	Q9NPH5	NOX4	0.89
	Alpha-2B adrenergic receptor	P18089	ADRA2B	0.89
	Prostaglandin G/H synthase 1	P23219	PTGS1	0.86
	Prostaglandin G/H synthase 2	P35354	PTGS2	0.86
KQCY6	Arachidonate 5-lipoxygenase	P09917	ALOX5	0.8
	Arachidonate 15-lipoxygenase	P16050	ALOX15	0.8
	Arachidonate 12-lipoxygenase	P18054	ALOX12	0.8
	Arachidonate 15-lipoxygenase B	O15296	ALOX15B	0.8
	Epidermis-type lipoxygenase 3	Q9BYJ1	ALOXE3	0.8
	Arachidonate 12-lipoxygenase	O75342	ALOX12B	0.75
	Aldo-keto reductase family 1 member B10	O60218	AKR1B10	0.71
	Aldose reductase	P15121	AKR1B1	0.71
	Aldo-keto reductase family 1 member B15	C9JRZ8	AKR1B15	0.71
	Estrogen receptor	P03372	ESR1	0.69
	Estrogen receptor beta	Q92731	ESR2	0.69
	Adenosine receptor A1	P30542	ADORA1	0.65
	Microtubule-associated protein tau	P10636	MAPT	0.63

	FAD-linked sulfhydryl oxidase ALR	P55789	GFER	0.61
	Adenosine receptor A3	P33765	ADORA3	0.61

Table S2 Relevant target genes of prostatitis

No.	Gene Symbol	Gene ID	Inference Score	No.	Gene Symbol	Gene ID	Inference Score
1	TP53	7157	68.8	278	PCNA	5111	33.82
2	FOLH1	2346	62.39	279	CLU	1191	33.81
3	RNASEL	6041	59.66	280	EGR1	1958	33.79
4	HPCAL1	3241	57.51	281	PAK1IP1	55003	33.72
5	ELAC2	60528	56.68	282	COL1A1	1277	33.7
6	KLF6	1316	54.42	283	IFIT1	3434	33.64
7	GNRH1	2796	53.1	284	GAL	51083	33.64
8	ER	246870	52.78	285	SRC	6714	33.63
9	IGF1R	3480	51.87	286	CDC25B	994	33.59
10	HOXB13	10481	51.87	287	P4HB	5034	33.58
11	MSR1	4481	51.03	288	COL18A1	80781	33.53
12	PRLR	5618	50.68	289	TNFRSF1A	7132	33.52
13	HMGB2	3148	50.55	290	PFKFB3	5209	33.48
14	PIM1	5292	50.49	291	MSMB	4477	33.48
15	AR	367	49.04	292	CCNT2	905	33.48
16	AKT1	207	48.09	293	BRCA2	675	33.48
17	CCND1	595	47.79	294	BAX	581	33.48
18	FOS	2353	47.42	295	STC2	8614	33.38
19	PSCA	8000	47.17	296	PBK	55872	33.38
20	COL3A1	1281	46.29	297	GJB2	2706	33.38
21	PGR	5241	46.27	298	MPP6	51678	33.37
22	MKLN1	4289	45.85	299	DDX60	55601	33.37
23	GADD45A	1647	45.81	300	CETN3	1070	33.37
24	CYP2E1	1571	45.77	301	CRHR2	1395	33.36
25	DNMT3B	1789	45.4	302	BUB1	699	33.28
26	EPHB2	2048	45.29	303	AP1G1	164	33.25
27	CEBPA	1050	45.18	304	HDAC8	55869	33.14
28	FADD	8772	45.08	305	CCDC86	79080	33.14
29	HNF1B	6928	44.76	306	SELENBP1	8991	33.13
30	GHR	2690	44.71	307	GAS6	2621	33.13
31	TGFB3	7043	44.53	308	EFNA1	1942	33.13
32	TP63	8626	44.5	309	FASLG	356	33.11
33	ODC1	4953	44.42	310	CYP11A1	1583	33.09
34	PIK3CA	5290	44.27	311	BAD	572	33.09
35	MXI1	4601	43.83	312	HSPB8	26353	33.08
36	FGF2	2247	43.82	313	KIF14	9928	33.04
37	CYP1A1	1543	43.81	314	FXYD3	5349	33.04
38	HIF1A	3091	43.67	315	ADIPOR1	51094	33.04
39	NKX3-1	4824	43.38	316	PGPEP1	54858	33.03
40	TMPRSS2	7113	43.34	317	GOLIM4	27333	33.03
41	EHBP1	23301	43.31	318	TIPARP	25976	32.93

42	TH	7054	43.28	319	PHTF2	57157	32.93
43	PDGFA	5154	43.16	320	NPM1	4869	32.93
44	LHB	3972	43.09	321	HDAC1	3065	32.93
45	TNFSF10	8743	43.03	322	BMP2K	55589	32.93
46	THOC1	9984	42.91	323	UGT2B1	71773	32.89
47	EGFR	1956	42.75	324	SAT1	6303	32.89
48	TSKU	25987	42.63	325	OLR139	293278	32.86
49	CYP1B1	1545	42.06	326	SAMD9	54809	32.82
50	CDK2	1017	42.06	327	RPL22L1	200916	32.82
51	PIK3R1	5295	42.02	328	NMI	9111	32.82
52	TIMP3	7078	41.98	329	MYOD1	4654	32.82
53	ABCG2	9429	41.82	330	INPP5A	3632	32.82
54	ABCC1	4363	41.76	331	HR	55806	32.82
55	CFLAR	8837	41.67	332	FANCC	2176	32.82
56	ANGPT1	284	41.56	333	ADAMTS2	9509	32.82
57	ECE1	1889	41.48	334	XDH	7498	32.8
58	CDKN1A	1026	41.47	335	IRS2	8660	32.79
59	ID1	3397	41.38	336	CDC6	990	32.79
60	BCL2L1	598	41.38	337	NAT2	10	32.75
61	CDK6	1021	41.29	338	MKI67	4288	32.75
62	CENPE	1062	41.18	339	IFNG	3458	32.75
63	ATF3	467	41.15	340	DHRS13A.2	447903	32.75
64	TUBB6	84617	40.89	341	STXBP6	29091	32.72
65	TGFB2	7042	40.85	342	SLC28A2	9153	32.72
66	MAF	4094	40.82	343	PARP12	64761	32.72
67	DNAJC3	5611	40.82	344	IPO4	79711	32.72
68	PRKCD	5580	40.77	345	HEG1	57493	32.72
69	NRIP1	8204	40.68	346	CYP24A1	1591	32.61
70	ACE	1636	40.62	347	PTEN	5728	32.6
71	PLA2G2A	5320	40.61	348	PAGE4	9506	32.56
72	CYP4B1	1580	40.61	349	HPC3	408259	32.53
73	SLC2A4	6517	40.58	350	MMP16	4325	32.52
74	PNPT1	87178	40.56	351	INSL3	3640	32.52
75	MYH14	79784	40.56	352	MIR205	406988	32.51
76	MAD1L1	8379	40.5	353	NFKB1	4790	32.49
77	CXCR4	7852	40.47	354	MIR21	406991	32.44
78	CDKN1B	1027	40.45	355	OPRL1	4987	32.43
79	CCND2	894	40.36	356	SRP9	6726	32.42
80	PCA3	50652	40.35	357	PCCA	5095	32.42
81	NBN	4683	40.15	358	EPB41L2	2037	32.42
82	PHLDA3	23612	40.14	359	ATP6V1B2	526	32.42
83	CYP3A23/3A1	25642	40.14	360	POR	5447	32.38
84	NR3C1	2908	40.11	361	CEBPB	1051	32.35
85	BIRC5	332	39.93	362	ENC1	8507	32.34

86	KLK2	3817	39.89	363	ADORA1	134	32.34
87	ARMCX3	51566	39.88	364	SLC44A4	80736	32.33
88	CTNNB1	1499	39.86	365	MND1	84057	32.33
89	FGFR4	2264	39.83	366	PLK1	5347	32.3
90	PRKCA	5578	39.82	367	NCOA1	8648	32.3
91	CCNB1	891	39.73	368	CLDN1	9076	32.3
92	SCNN1A	6337	39.71	369	CKB	1152	32.3
93	PLAU	5328	39.51	370	CDK4	1019	32.3
94	SLC39A11	201266	39.5	371	UBE2C	11065	32.25
95	EDN1	1906	39.31	372	MAPK14	1432	32.25
96	TCF21	6943	39.14	373	PSPC1	55269	32.23
97	IGF1	3479	39.12	374	ITPR1	3708	32.21
98	PHB2	11331	39.03	375	SOX9	6662	32.17
99	ELOVL1	64834	39.03	376	ADGRG2	10149	32.15
100	FSHB	2488	38.92	377	FGD4	121512	32.13
101	AMH	268	38.91	378	ATXN3	4287	32.13
102	LPIN1	23175	38.84	379	ANGPTL2	23452	32.13
103	LMNB1	4001	38.84	380	PARP1	142	32.12
104	PTPRN2	5799	38.8	381	BID	637	32.09
105	JUN	3725	38.8	382	MIR145	406937	32.06
106	NFIL3	4783	38.78	383	MMP2	4313	32.04
107	GPATCH4	54865	38.58	384	ENPP2	5168	32.04
108	ATP1B1	481	38.57	385	ERBB2	2064	32.01
109	CAT	847	38.5	386	ZFHX3	463	32
110	GJA1	2697	38.49	387	SLC1A2	6506	32
111	MCL1	4170	38.43	388	GSN	2934	32
112	KLK3	354	38.41	389	HPC14	1E+08	31.98
113	PADI2	11240	38.37	390	PCAP	7834	31.95
114	VEGFA	7422	38.26	391	PARP9	83666	31.95
115	AHR	196	38.26	392	MACF1	23499	31.95
116	TOP2A	7153	38.22	393	CCNA2	890	31.95
117	DUSP1	1843	38.22	394	AGR2	10551	31.95
118	F2R	2149	38.2	395	KLF4	9314	31.92
119	VCPKMT	79609	38.19	396	BMP4	652	31.91
120	KDELR3	11015	38.16	397	EPHA1	2041	31.87
121	CCN2	1490	38.08	398	CCT7	10574	31.87
122	STAT1	6772	38.02	399	SLC16A1	6566	31.79
123	STAT3	6774	37.99	400	CYP2A4	13086	31.79
124	ESR1	2099	37.97	401	FZD5	7855	31.78
125	DNMT3A	1788	37.96	402	HSD17B12	51144	31.77
126	AQP1	358	37.96	403	TLR4	7099	31.72
127	KISS1	3814	37.87	404	CKAP4	10970	31.7
128	MBP	4155	37.76	405	STARD13	90627	31.69
129	MMP14	4323	37.71	406	AKAP1	8165	31.69

130	MED11	400569	37.69	407	MIR221	407006	31.67
131	HOPX	84525	37.67	408	SDR39U1	56948	31.64
132	CD68	968	37.67	409	HPC4	408260	31.63
133	FADS2	9415	37.62	410	AURKA	6790	31.63
134	KRT19	3880	37.57	411	GAA	2548	31.61
135	TSC22D3	1831	37.53	412	DMBT1	1755	31.61
136	GOT1	2805	37.53	413	IGFBP5	3488	31.59
137	MDM2	4193	37.52	414	HPC10	1E+08	31.57
138	CCNE1	898	37.5	415	SRD5A2	6716	31.52
139	TK1	7083	37.48	416	RUNX1T1	862	31.52
140	JUND	3727	37.43	417	ACPP	55	31.52
141	UBQLN4	56893	37.24	418	TNFRSF12A	51330	31.51
142	IL1R1	3554	37.21	419	RSRC2	65117	31.44
143	CXCL10	3627	37.18	420	PPARGC1A	10891	31.44
144	STAR	6770	37.1	421	P2RY1	5028	31.44
145	CYP17A1	1586	37.05	422	NTRK2	4915	31.44
146	ZYX	7791	37.04	423	FAM13A	10144	31.44
147	STAT2	6773	37.04	424	TRPV6	55503	31.36
148	HSDL2	84263	37.04	425	SLC39A4	55630	31.36
149	GSTP1	2950	37.04	426	RCAN2	10231	31.36
150	CYP1A2	1544	37.02	427	CASP6	839	31.36
151	GADD45G	10912	36.94	428	CASP2	835	31.36
152	ARNT	405	36.94	429	MYC	4609	31.35
153	POLK	51426	36.87	430	EIF2S2	8894	31.35
154	ATP2A2	488	36.86	431	HSD3B1	3283	31.32
155	RARA	5914	36.81	432	RGD1559459	501907	31.3
156	HRAS	3265	36.77	433	DHX9	1660	31.29
157	FOXA1	3169	36.71	434	KLK5L	408211	31.27
158	ARPC1B	10095	36.71	435	FAM43A	131583	31.27
159	FOXO3	2309	36.65	436	FKBP5	2289	31.25
160	LXN	56925	36.63	437	ARG1	383	31.25
161	TFF1	7031	36.61	438	HPC5	619402	31.22
162	MSMO1	6307	36.61	439	UGT2B17	7367	31.2
163	EGF	1950	36.6	440	TNF	7124	31.2
164	DDIT3	1649	36.55	441	SYNJ2	8871	31.2
165	SMC2	10592	36.54	442	RAD23A	5886	31.2
166	SRD5A1	6715	36.52	443	GNAO1	2775	31.2
167	SREBF1	6720	36.48	444	CEMIP2	23670	31.2
168	PTK2	5747	36.48	445	PHKG1	5260	31.19
169	ID3	3399	36.44	446	MAPK11	5600	31.19
170	NUP133	55746	36.42	447	TANGO2	128989	31.17
171	RAD51	5888	36.4	448	LGALS3	3958	31.17
172	GSR	2936	36.39	449	AKR1CL	70861	31.17
173	PYGM	5837	36.38	450	HPCQTL19	347747	31.15

174	BRCA1	672	36.36	451	CYP2C9	1559	31.14
175	SMC4	10051	36.31	452	TSPAN8	7103	31.12
176	CD82	3732	36.27	453	TRP53	22059	31.12
177	SNW1	22938	36.24	454	PRKD1	5587	31.12
178	ATAD2	29028	36.23	455	SQLE	6713	31.1
179	ALAS2	212	36.16	456	TGFBR2	7048	31.06
180	NR5A1	2516	36.15	457	TNNC1	7134	31.04
181	ESRRA	2101	36.15	458	SLC15A2	6565	31.04
182	VIM	7431	36.13	459	PARP14	54625	31.04
183	RPS6KA3	6197	36.08	460	GDF10	2662	31.04
184	IGF2	3481	36.08	461	HSPH1	10808	31.02
185	GLI1	2735	36.08	462	MSMP	692094	31.01
186	DBH	1621	36.08	463	TGFA	7039	30.99
187	SMTNL2	342527	36.05	464	PFN2	5217	30.97
188	HSP90B1	7184	36.05	465	PKP2	5318	30.96
189	IL1B	3553	36.02	466	CLCN1	1180	30.96
190	LY6E	4061	36	467	POC5	134359	30.95
191	MET	4233	35.97	468	MORN2	729967	30.95
192	CASP9	842	35.96	469	KCNF1	3754	30.94
193	SPAG5	10615	35.93	470	CDC25C	995	30.92
194	GPX3	2878	35.93	471	IL6	3569	30.9
195	BDNF	627	35.9	472	CREB1	1385	30.9
196	DNMT1	1786	35.89	473	SLC38A4	55089	30.89
197	MRPS27	23107	35.88	474	COX5B	1329	30.89
198	MMD2	221938	35.88	475	ANP32E	81611	30.89
199	PCLAF	9768	35.86	476	CYP3A2	266682	30.85
200	CKS1B	1163	35.86	477	CYP19A1B	60640	30.82
201	CDH1	999	35.81	478	BCL2	596	30.82
202	ERG	2078	35.76	479	TNS3	64759	30.81
203	SPDEF	25803	35.71	480	OAS1	4938	30.81
204	ESPNP	284729	35.64	481	MGST1	4257	30.81
205	APAF1	317	35.63	482	STEAP2	261729	30.79
206	IRS1	3667	35.6	483	S100A9	6280	30.77
207	CYP19A1	1588	35.59	484	SIAH2	6478	30.74
208	COL6A3	1293	35.57	485	EFHD1	80303	30.74
209	ATP2B1	490	35.57	486	CPEB2	132864	30.74
210	RAB12	201475	35.54	487	ARHGAP18	93663	30.74
211	PWP2	5822	35.54	488	HIST1H2BO	8348	30.72
212	HNRNPH1	3187	35.5	489	CYBA	1535	30.71
213	CCN1	3491	35.45	490	SPSB1	80176	30.67
214	PCBP3	54039	35.38	491	S100G	795	30.67
215	ABCD3	5825	35.37	492	S100A8	6279	30.67
216	ACTA2	59	35.27	493	IFIH1	64135	30.67
217	MYO18A	399687	35.22	494	HBEGF	1839	30.64

218	CAMK2D	817	35.17	495	FSHR	2492	30.61
219	INSIG1	3638	35.14	496	TIA1	7072	30.6
220	CPE	1363	35.1	497	NUPR1	26471	30.6
221	NUP107	57122	35.07	498	CYBB	1536	30.6
222	LHCGR	3973	35.04	499	CRIP2	1397	30.6
223	CTSD	1509	35	500	STARD4	134429	30.59
224	NOS2	4843	34.97	501	PADI1	29943	30.53
225	RELA	5970	34.95	502	ENAM	10117	30.53
226	TIMP2	7077	34.93	503	DLC1	10395	30.53
227	FUCA2	2519	34.92	504	CLMN	79789	30.53
228	ASB13	79754	34.92	505	LRRFIP1	9208	30.52
229	CGA	1081	34.91	506	FANCI	55215	30.52
230	MUC1	4582	34.84	507	EIF2S3	1968	30.52
231	GSK3B	2932	34.82	508	CDH11	1009	30.52
232	C3	718	34.73	509	MAFF	23764	30.47
233	GBP2	2634	34.66	510	VTG1	559475	30.46
234	ECT2	1894	34.66	511	KNSTRN	90417	30.46
235	ZP3	7784	34.64	512	INS1	16333	30.45
236	TWNK	56652	34.63	513	PXN	5829	30.39
237	WARS	7453	34.6	514	HPC7	1E+08	30.39
238	KLHL24	54800	34.6	515	ABHD2	11057	30.39
239	SLC45A3	85414	34.58	516	EPHA4	2043	30.38
240	SRPRA	6734	34.49	517	MIR146A	406938	30.36
241	CBFA2T3	863	34.49	518	WDR60	55112	30.35
242	SLC6A6	6533	34.48	519	MIR222	407007	30.34
243	NFKBIA	4792	34.48	520	CRYAB	1410	30.34
244	DLGAP5	9787	34.48	521	SDK1	221935	30.33
245	RXRA	6256	34.38	522	EIF3K	27335	30.33
246	CD44	960	34.38	523	TM4SF1	4071	30.32
247	WWC2	80014	34.35	524	HAO2	51179	30.32
248	DGKH	160851	34.35	525	DTL	51514	30.32
249	VCAM1	7412	34.34	526	PGRMC1	10857	30.31
250	IGFBP6	3489	34.3	527	RRM2	6241	30.3
251	NR4A1	3164	34.26	528	IGFBP1	3484	30.3
252	SLC29A1	2030	34.24	529	GPX1	2876	30.3
253	PROM1	8842	34.24	530	CHEK2	11200	30.3
254	ESR2	2100	34.22	531	ALB	213	30.3
255	LAMC2	3918	34.19	532	CDH2	1000	30.27
256	AHNAK	79026	34.19	533	PSRC1	84722	30.25
257	HMOX1	3162	34.14	534	PAWR	5074	30.25
258	IL2	3558	34.1	535	NOLC1	9221	30.25
259	RORA	6095	34.02	536	MX2	4600	30.25
260	MUC5B	727897	33.97	537	ELF3	1999	30.25
261	TGFB1	7040	33.96	538	TLR2	7097	30.24

262	RALGAPA1	253959	33.96	539	SOCS3	9021	30.21
263	PPARG	5468	33.96	540	HPC6	1E+08	30.21
264	MRPL33	9553	33.96	541	HPC15	1E+08	30.21
265	MEN1	4221	33.96	542	EPOR	2057	30.18
266	IFT88	8100	33.96	543	GH	14599	30.16
267	GUSB	2990	33.96	544	SLC7A6OS	84138	30.15
268	FAM110C	642273	33.96	545	LAS1L	81887	30.15
269	NQO1	1728	33.94	546	ZBTB22	9278	30.14
270	CASP8	841	33.94	547	PCGEM1	64002	30.12
271	HSD17B1	3292	33.91	548	SP1	6667	30.11
272	CD80	941	33.91	549	ME1	4199	30.08
273	CD38	952	33.91	550	CYCS	54205	30.06
274	MCM7	4176	33.9	551	OPTN	10133	30.05
275	BCL3	602	33.9	552	MYH10	4628	30.05
276	SERPINE1	5054	33.88	553	ITPR3	3710	30.05
277	MTHFD2	10797	33.85	554	MIR34A	407040	30.01

Table S3 The information of merging gene

No.	Gene	Gene ID	Gene Name
1	XDH	7498	xanthine dehydrogenase(XDH)
2	CYP1B1	1545	cytochrome P450 family 1 subfamily B member 1(CYP1B1)
3	HSD17B1	3292	hydroxysteroid 17-beta dehydrogenase 1(HSD17B1)
4	ESR1	2099	estrogen receptor 1(ESR1)
5	ABCC1	4363	ATP binding cassette subfamily C member 1(ABCC1)
6	ESR2	2100	estrogen receptor 2(ESR2)
7	CYP1A2	1544	cytochrome P450 family 1 subfamily A member 2(CYP1A2)
8	NQO1	1728	NAD(P)H quinone dehydrogenase 1(NQO1)
9	ADORA1	134	adenosine A1 receptor(ADORA1)
10	AHR	196	aryl hydrocarbon receptor(AHR)

Table S4 Details of enriched pathways

Term	Database	ID	Input number	Background number	P-Value	Corrected P-Value
Caffeine metabolism	KEGG PATHWAY	hsa00232	2	5	1.19E-06	3.36E-05
omega-hydroxylase P450 pathway	Gene Ontology	GO:0097267	2	9	3.13E-06	6.38E-05
ERBB signaling pathway	Gene Ontology	GO:0038127	3	145	5.95E-06	0.0001
epoxygenase P450 pathway	Gene Ontology	GO:0019373	2	18	1.08E-05	0.000161
Tryptophan metabolism	KEGG PATHWAY	hsa00380	2	40	4.87E-05	0.000493
enzyme linked receptor protein signaling pathway	Gene Ontology	GO:0007167	4	942	5.96E-05	0.000577
Prolactin signaling pathway	KEGG PATHWAY	hsa04917	2	72	0.000152	0.001114
Estrogen signaling pathway	KEGG PATHWAY	hsa04915	2	99	0.000284	0.001809
Sphingolipid signaling pathway	KEGG PATHWAY	hsa04071	2	121	0.00042	0.002448
hormone-mediated signaling pathway	Gene Ontology	GO:0009755	2	214	0.001284	0.005838
Metabolic pathways	KEGG PATHWAY	hsa01100	3	1243	0.003121	0.010098
adenosine receptor signaling pathway	Gene Ontology	GO:0001973	1	12	0.003264	0.010218
vascular endothelial growth factor signaling pathway	Gene Ontology	GO:0038084	1	24	0.006269	0.01554
purinergic receptor signaling pathway	Gene Ontology	GO:0035587	1	31	0.008018	0.017868
Thyroid hormone signaling pathway	KEGG PATHWAY	hsa04919	1	118	0.029526	0.044206
G-protein coupled receptor signaling pathway	Gene Ontology	GO:0007186	2	1204	0.035176	0.050369
cGMP-PKG signaling pathway	KEGG PATHWAY	hsa04022	1	167	0.041453	0.057463
cAMP signaling pathway	KEGG PATHWAY	hsa04024	1	199	0.049172	0.065465
Purine metabolism	KEGG PATHWAY	hsa00230	1	176	0.04363	0.05956

Table S5 Gradient elution program

Time（min）	A%	B%	Curve
0.00	98.0	2.0	Initial
8.00	60.0	40.0	6
10.00	2.0	98.0	6
12.00	98.0	2.0	6
14.00	98.0	2.0	6

Table S6 Markerlynx Software parameter settings

Parameter	ESI(+)	ESI(-)
Function	1	1
Analysis type	Peak Detection	Peak Detection
Initial/Final Retention Time	0.00/10.00min	0.00/10.00min
Low/High Mass	50.00/1200.00 Da	50.00/1200.00 Da
XIC Window (Da)	0.05	0.05
Use relative retention time	NO	NO
Peak width at 5% Height (seconds)	1.00	1.00
Peak-to-Peak Baseline Noise	0.00	0.00
Apply Smoothing	NO	NO
Marker Intensity threshold (counts)	100	100
Mass window	0.05 Da	0.05 Da
Retention time window	0.10 min	0.10 min
Noise elimination level	6.00	6.00
Deisotope data	YES	YES
Replicate% Minimum	0.00	0.00

Table S7 Structures of active compounds

No	ID	Structure	No	ID	Structure
1	KQCR-1	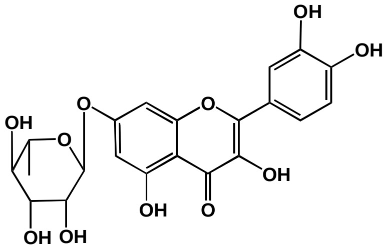 Quercetin-7-O-α-L- rhamnopyranoside	2	KQCR-2	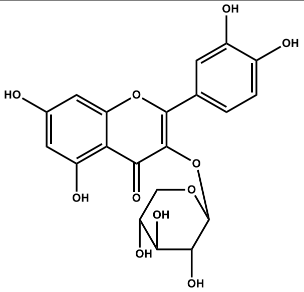 Quercetin-3-O-α-L-arabinopyranoside
3	KQCR-5	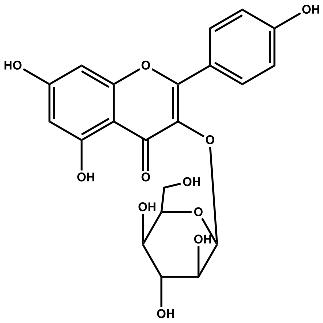 Kaempferol-3-O-β-D-glucopyranoside	4	KQCR-6	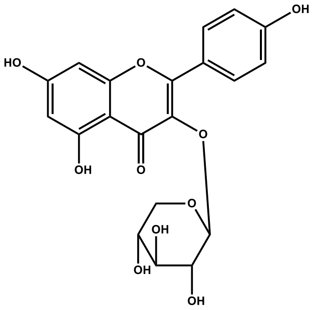 Kaempferol-3-O-β-D-xylopyranoside
5	KQCR-9	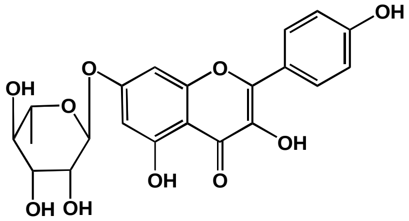 Kaempferol-3-O-α-L- rhamnopyranoside	6	KQCR-11	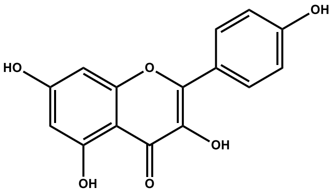 Kaempferol
7	KQCR-12	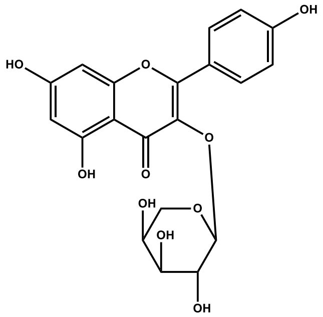 Kaempferol-3-O-α-L-arabonipyranoside	8	KQCY-6	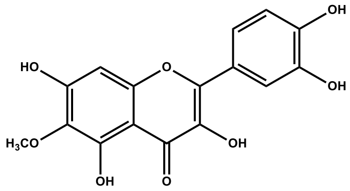 Patuletin
